# Reliability, bias, and computational cost of estimating the Bayes factor using bridge sampling and the Savage–Dickey density ratio

**DOI:** 10.3758/s13428-026-03087-w

**Published:** 2026-06-22

**Authors:** Klaus Oberauer, Philipp Musfeld, Frederik Aust

**Affiliations:** 1 Department of Psychology, University of Zurich, Binzmühlestrasse 14/22, 8050 Zurich, Switzerland; 2 Department of Psychology, University of Cologne, Cologne, Germany

**Keywords:** Bayes factor, Bridge sampling, Bavage-dickey density ratio

## Abstract

Bayes factors often require numerical estimation because closed-form solutions are unavailable. In six simulation studies, we explored the reliability, bias, and computational cost of two easy-to-use and broadly applicable methods: bridge sampling and the Savage–Dickey density ratios, based on Gaussian, logspline, and spline-smoothed kernel density approximations of the posterior distribution, as well as conditional marginal density estimation. In generalized linear mixed effect models for normally and binomially distributed data, we explore the effects of the (1) number of MCMC samples from the posterior, (2) size of effects or magnitude of the Bayes factor, (3) number of participants, and (4) number of model parameters. Our findings suggest that, with enough MCMC samples, both methods yield reliable and accurate estimates across a wide range of conditions. However, with many model parameters, bridge sampling becomes computationally expensive and can be unreliable. In contrast, the Savage–Dickey density ratio scales well, remaining computationally efficient and reliable, even with many model parameters. However, Savage–Dickey density ratio requires careful consideration of posterior density estimation to mitigate bias while limiting the variability of Bayes factor estimates. We provide practical recommendations to guide researchers in selecting the most suitable estimation method for their applications.

## Introduction

Testing whether an effect of an independent variable on a dependent variable exists (${\mathcal{H}}_{1}$) or not (${\mathcal{H}}_{0}$) is the most frequent hypothesis test in psychology and other social sciences. In light of the conceptual problems of classical null-hypothesis significance testing (Wagenmakers, 2007), many researchers are turning to Bayesian hypothesis testing as the alternative with a more sound foundation in statistical theory. In the Bayesian framework, testing ${\mathcal{H}}_{1}$ against ${\mathcal{H}}_{0}$ comes down to comparing two statistical models, ${\mathcal{M}}_{1}$ and ${\mathcal{M}}_{0}$, which differ only in that ${\mathcal{M}}_{1}$ includes a free parameter for the effect in question, whereas in ${\mathcal{M}}_{0}$ that parameter is fixed to a value reflecting no effect of the independent variable, such as chance performance or zero.

The gold standard for Bayesian model comparison is to use the Bayes factor (BF) as a measure of the relative evidence in favor of one model over the other. The BF follows directly from Bayes’ theorem and is the ratio of the marginal likelihoods of the two models, ${\mathrm{B}\mathrm{F}}_{10}=p\left({\boldsymbol{y}} | {\mathcal{M}}_{1}\right)/p\left({\boldsymbol{y}} | {\mathcal{M}}_{0}\right)$. The marginal likelihood of a model, $p\left({\boldsymbol{y}} | {\mathcal{M}}_{i}\right)$, is the average likelihood of the observed data ${\boldsymbol{y}}$ across all possible model parameter values, weighted by their prior probability. In other words, the marginal likelihood is a measure of a model’s prior predictive accuracy. Hence, ${\mathrm{B}\mathrm{F}}_{10}$ indicates whether ${\mathcal{M}}_{1}$ or ${\mathcal{M}}_{0}$ more accurately anticipates the observed data, and by how much. For example, a ${\mathrm{B}\mathrm{F}}_{10}=3$ means that ${\mathcal{M}}_{1}$ predicts the observed data 3 times better than ${\mathcal{M}}_{0}$. This is why ${\mathrm{B}\mathrm{F}}_{10}$ is sometimes referred to as the *evidence* for ${\mathcal{M}}_{1}$ relative to ${\mathcal{M}}_{0}$. The direction of the comparison is indicated by the index, hence ${1/\mathrm{B}\mathrm{F}}_{10}={\mathrm{B}\mathrm{F}}_{01}$ gives the evidence for ${\mathcal{M}}_{0}$ relative to ${\mathcal{M}}_{1}$.

The main limiting factor on using BF for hypothesis testing is practical: They are hard to compute. Analytical solutions exist only for relatively simple cases (Rouder et al., 2012; Rouder et al., 2009), so that researchers mostly have to resort to numeric estimation of BFs based on Markov chain Monte Carlo (MCMC) samples from the posterior distribution. Several estimation methods exist that differ in bias, reliability, generality, and ease of use, such as the Savage–Dickey density ratio (Dickey, 1971; Dickey & Lientz, 1970), Reversible Jump MCMC (Green, 1995), Chib’s method (Chib, 1995), bridge sampling (Meng & Wong, 1996), path sampling (Gelman & Meng, 1998), and others (for a review see Llorente et al.,  2023). A few general considerations reduce the number of available estimation methods. First, we believe most researchers place a premium on ease of use. Second, in our experience, nested model comparisons and specifically comparisons that test individual parameters in (hierarchical) generalized linear models are the most common statistical hypothesis tests in psychology and other social sciences. Choosing among the methods that fit this description requires researchers to consider the reliability of BF estimates (i.e., how much the estimates vary from one estimation to the next on the same data), their bias, and their computation costs. Here we report six simulations designed to help practical researchers to choose an easy-to-use BF estimation method for nested model comparisons in (hierarchical) generalized linear models.

We investigate two broadly applicable methods of estimating BFs^1^, bridge sampling (Gronau et al., 2020; Gronau et al., 2018) and the Savage–Dickey density ratio (Wagenmakers et al., 2010). Bridge sampling is a simulation-based method for approximating the marginal likelihood of a model—again, Bayes factors are simply the ratio of competing models’ marginal likelihoods. Bridge sampling is a general method in the sense that it is not restricted to comparisons of nested models, where the constrained model results from removing the parameter of interest θ (e.g., a condition difference) from the encompassing model, but is also applicable to non-nested model comparisons (Gronau et al., 2017; Meng & Wong, 1996). Unfortunately, this generality comes at a cost: Bridge sampling can be computationally expensive. Estimation of the marginal likelihood requires that one obtain a relatively large number of samples from the joint posterior distribution, which are then submitted to the bridge sampling algorithm. Both steps can be computationally demanding and are required for all competing models.

The Savage–Dickey density ratio is a simpler but less flexible method to calculate the Bayes factor. Instead of estimating each models’ marginal likelihood separately, the Bayes Factor is calculated directly as the ratio of the prior and the posterior density of the parameter of interest θ (e.g., a condition difference) at a theoretically interesting value (e.g., chance performance or zero; Wagenmakers et al., 2010). Often this requires the *estimation* of the marginal posterior distribution of θ because it is not analytically available. The Savage–Dickey density ratio is computationally less expensive than bridge sampling because (1) only posterior samples from the encompassing model are required^2^ and (2) approximating the marginal posterior distribution for the parameter of interest θ is less computationally demanding than the bridge sampling algorithm. However, this efficiency again comes at a cost: The Savage–Dickey density ratio is limited to nested model comparisons (and prior distributions that assign a finite, non-zero density to the test value; see Heck (2019) for other important caveats). Moreover, it is difficult in practice to use the Savage–Dickey density ratio to compare models that differ in a more than one parameter. A common example are omnibus tests of the overall effect of a categorical predictor with more than two levels, where that effect is represented by *n*−1 parameters (for *n* levels) in the model.^3^

To the best of our knowledge, there is limited guidance on choosing between bridge sampling and the Savage–Dickey density ratio when both methods are applicable. However, several factors are known to affect the reliability and bias of these methods. By reliability, we mean the variance of repeated estimates of the BF with the same method using the same data. In several examples of generalized linear models, bridge sampling estimates of the BF have been found to be unbiased and reliable compared to reversible jump MCMC, importance sampling, and Chib’s method (Meng & Schilling, 1996; Sinharay & Stern, 2005). The reliability of the estimates depends on the number of MCMC samples (Gronau, Wagenmakers, Heck, & Matzke, 2019), but beyond rules of thumb (“about an order of magnitude more posterior samples than estimation”, p. 12, Gronau et al., 2018), the practical researcher is left to their own devices.

In Savage–Dickey density ratios, the bias and variance of the estimates depends on the method used to approximate the posterior density and its ability to match the true distribution (Morey et al., 2011). Two popular methods are logspline density estimation (Makowski et al., 2019; Morey et al., 2011; Wagenmakers et al., 2010) and approximating the posterior by a normal distribution (Morey et al., 2011). The latter approach appeals to the Bayesian central limit theorem, which posits that asymptotically marginal posterior distributions will be normally distributed (Bernardo & Smith, 2000; cf. Bernstein-von Mises theorem). Morey et al. (2011) compared both methods in a simulation study for a simple *t* test. They found that assuming a normal distribution yields more reliable estimates than logspline density estimation, but reliabilities of both estimates can increase with the number of MCMC samples used for the density estimation. In finite samples, both methods yielded biased estimates—in particular when ${\mathrm{BF}}_{10}$ was large—but for different reasons. Assuming a normal distribution yielded biased estimates because the normal distribution misfitted the true posterior distribution. With more data, the bias decreased, as predicted by the Bayesian central limit theorem; increasing the number of MCMC samples did not affect bias. In contrast, logspline density estimation yielded biased estimates because it is difficult to estimate the density in the tail of the distribution where few MCMC samples are available. The logspline method addresses this problem by assuming that the density decreases exponentially when few samples are available (Kooperberg & Stone, 1991). Hence, increasing the number of MCMC samples increases reliability and decreases bias of the BF estimates. However, compared to the normal approximation, logspline density estimates performed so poorly overall that Morey et al. (2011) did not consider them in subsequent simulations. Based on these findings, an informative comparison of bridge sampling and the Savage–Dickey density ratio should compare their bias and reliability and how they are affected by the number of MCMC samples, the magnitude of BF (e.g., effect size), and the sample size (which affects the normality of the posterior). In addition, the number of model parameters is also likely to be a relevant factor because it strongly influences the difficulty of estimating the posteriors.

Morey et al. (2011) also considered a third method for computing Savage–Dickey density ratios, conditional marginal density estimation (Chib, 1995; Gelfand & Adrian, 1990), and show that it is a more reliable and less biased estimation method than the logspline and normal approximations of the posterior probability density. Unfortunately, this method is only applicable when the conditional posterior distribution $p\left(\theta \right|\phi , {\boldsymbol{y}}, {\mathcal{M}}_{1})$ is known, which is usually not the case in hierarchical generalized linear models. Therefore, we do not consider conditional marginal density estimation in most of our simulations. We include it as a comparison standard in Simulation 6.

The remainder of this paper is organized as follows. We present six simulation studies that compare Bayes factors estimated using the Savage–Dickey density ratio and the bridge sampling algorithm (for an overview, see Table 1). Simulations 1–4 explore bias and reliability of bridge sampling and the Savage–Dickey density ratio for hierarchical generalized linear models, which we apply to simulations of typical experimental designs in psychology. Simulation 1 varies the number of posterior samples and compares the estimation methods for normally and binomially distributed data. Simulation 2 varies the effect size and thereby the magnitude of the to-be-estimated BF. Simulations 3 and 4 explore the effect of model complexity. In Simulation 3, we increase the number of to-be-estimated parameters, while keeping the sample size rather small (*N =* 20). In Simulation 4, we additionally increased the sample size (*N* = 100), which further increases the number of to-be-estimated parameters due to the estimation of random participant effects. Simulations 5 and 6 then explore some noticeable findings of our first five simulations in more depth. Simulation 5 compares three density estimators, widely used to obtain Savage–Dickey density ratios, by varying the tail probability of the test value and the number of posterior samples. In a simplified model for which analytic BFs are available, Simulation 6 jointly varies the magnitude of the to-be-estimated BF and the number of model parameters. We compare bridge sampling to Savage–Dickey density ratios based on the three density estimators explored in Simulation 5 and assess their reliability, bias, and computational cost. Finally, we summarize our findings and discuss the factors that should be considered when choosing between the Savage–Dickey density ratio and bridge sampling.

**Table 1:** Overview of findings

Simulation	Question	Conclusion
1	Effect of number of MCMC samples	Reliability of bridge sampling and Savage–Dickey BF estimates increases with number of samples
2	Effect of the size of experimental effects	The variability of BFs scales with the mean BF. It is tolerable for all effect sizes
3	More complex experimental design	Reliability of BF estimates is not compromised by more complex designs
4	Larger sample size, with more parameters to estimate	Reliability of bridge sampling BF estimates suffers, and becomes worryingly poor. Savage–Dickey estimates still have tolerable variability.
5	Comparison of different methods for computing the Savage–Dickey ratio	For normally distributed posteriors, the normal approximation is less variable and less biased than the logspline density estimation, which is less variable than spline-smoothed kernel density estimation. The normal approximation risks substantial bias when the posterior distributions deviate strongly from normality. Logspline density estimation can fail catastrophically when the number of MCMC samples is too *high*
6	Effect of magnitude of evidence for an effect, and of the number of model parameters	Variability of the Savage–Dickey BF estimates increases only with the strength of evidence for an effect, whereas the variability of bridge-sampling estimates increases only with the number of model parameters. Bridge sampling and CMDE remain unbiased across all conditions, whereas bias of all other Savage–Dickey approximation methods increases with stronger evidence for an effect

## Simulation 1: Varying MCMC sample size

For both bridge sampling and Savage–Dickey density ratio, reliability of BF estimates is limited by the number of MCMC samples (Gronau et al., 2019; Morey et al., 2011). Outside of very simple models and small data sets, however, the number of MCMC samples is limited by the available computational resources and computing time. Hence, researchers face a trade-off: While larger MCMC sample sizes yield more reliable BF estimates, they demand greater computational resources and time. This trade-off may be different for bridge sampling and the Savage–Dickey density ratio. To quantify these relationships, we conducted a simulation study examining how MCMC sample size affects BF estimate reliability in hierarchical generalized linear models for typical experimental designs.

### Simulation 1a: Normally distributed dependent variable

We ran simulations for a typical experimental design in psychology, involving one between- and one within-subjects manipulation as independent variables (IV). We considered two kinds of dependent variables: (1) Normally distributed, because the assumption of normality is pervasive and underlies the classical frequentist ANOVA and the default Bayesian ANOVA (Rouder et al., 2012)^4^, and (2) binomially distributed, because dichotomous outcomes, like two-alternative forced-choice or responses coded as correct/incorrect, are also common in experimental psychology.

The simulated experiment has two IVs. IV 1 is a continuous variable varied within-subjects with five levels; IV 2 is a categorical variable varied between subjects with two levels (i.e., two groups). Each IV had a true effect size of *d* = 0.25, but there was no interaction. Effect sizes were unstandardized and defined as the deviation of condition means from the grand mean, rather than as the difference between condition means. Data were simulated for *N* = 20 subjects per group, and *n* = 150 responses per design cell, according to the following model:

$$\begin{array}{l}{y}_{ij}\sim N({m}_{i}+{d}_{1,i}{X}_{1,i,j}+{d}_{2}{X}_{2,i},\zeta )\\ {m}_{i}\sim N(0,{\sigma}_{m})\\ {d}_{1,i}\sim N({d}_{1},{\sigma}_{{d}_{1}})\end{array}$$

Here, *y*_*ij*_ is the response of subject *i* in trial *j*. *X*_*1,i,j*_ is the level of IV 1 of trial* j* for subject *i*; *X*_*2,i*_ is the level of IV 2 of subject *i*; *d*_1_ and *d*_2_ are the fixed effect sizes of IV 1 and IV 2, respectively. IV 1 was *z*-standardized (resulting in levels – 1.265, – 0.632, 0, 0.632, 1.265); IV 2 was contrast-coded (– 1 vs. 1). Each subject has an individual mean value of the dependent variable, *m*_*i*_, and an individual effect size of IV 1, *d*_*1,i*_. These random effects are normally distributed with standard deviation ${\sigma}_{m}={\sigma}_{{d}_{1}}=0.3$. Trial-by-trial variability was modeled by normally distributed error with standard deviation ξ = 1.0.

We simulated ten data sets and estimated hierarchical linear models assuming normally distributed residuals with the brms package in R (Bürkner, 2025; R Core Team, 2024). The encompassing model matched the data-generating process, but additionally assumed an interaction effect between IV1 and IV2: y ~ iv1 * iv2 + (1 + iv1 || subj) (Oberauer, 2022).^5^ All estimated models also included random participant effects on all within-subject parameters.^6^ We used Cauchy priors with scale 0.5 for the fixed effects, and Gamma priors with shape 1 and rate 0.01 for the random effects. Each model was run with eight chains, and we varied the number of MCMC samples per chain (5000 vs. 10,000 samples per chain). The first 1000 samples of each chain were used as HMC warmup and discarded. This results in 32,000 or 72,000 samples from the posterior distribution. The number of samples we investigated far exceeds the default number of samples per chain in brms (4000) because whereas the default number is appropriate for parameter estimation, it is likely to be insufficient for BF estimation.

For each simulated data set, we estimated the evidence for each effect (BF_10_) via bridge sampling (Gronau et al., 2020; Gronau et al., 2018) and the Savage–Dickey density ratio (Wagenmakers et al., 2010). For the bridge sampling method, we estimated BFs through a series of model comparisons. For each effect, we compared the full model to a model in which the fixed effect of interest – but not the corresponding random effect – was removed. BFs were estimated using the bayes_factor() function in brms, which in turn calls the bridge_sampler() function from the bridge-sampling R-package that estimates the marginal likelihoods of each model (Gronau et al., 2018). We used all default settings, which set the bridge sampling method to “normal” (as compared to “Warp-III”) and runs the bridge sampler for a maximum of 1000 iterations.

For the Savage–Dickey density ratio, we determined the prior density at zero from the analytical density of the Cauchy prior. For the corresponding posterior density, we assumed the posterior distribution to be approximately normally distributed (Bayesian central limit theorem; Bernardo & Smith, 2000) and therefore we evaluated the density of a normal distribution at zero with mean and standard deviation equal to the posterior mean and the standard deviation for the effect in question (e.g., the main effect of IV 1). Hence, the estimated BF_10_ in favor of the effect Xi is

$$\begin{array}{cl}B{F}_{10}\left({X}_{i}\right)=\frac{Cauchy\left(0;\mathrm{0,0.5}\right)}{Normal\left(0;\mu \left({d}_{i}\right),\sigma \left({d}_{i}\right)\right)}\\ \mu \left({d}_{i}\right)=\frac{1}{m}\sum\limits_{k=1}^{m}{d}_{i,k};\\ \sigma \left({d}_{i}\right)=\sqrt{\frac{1}{m-1}{\sum\limits_{k=1}^{m}\left({d}_{i,k}-\mu \left({d}_{i}\right)\right)}^{2}}\end{array}$$

where *d*_*i,k*_ is the *k*th sample from the MCMC sample of effect size *d*_*i*_.

**Fig. 1:**
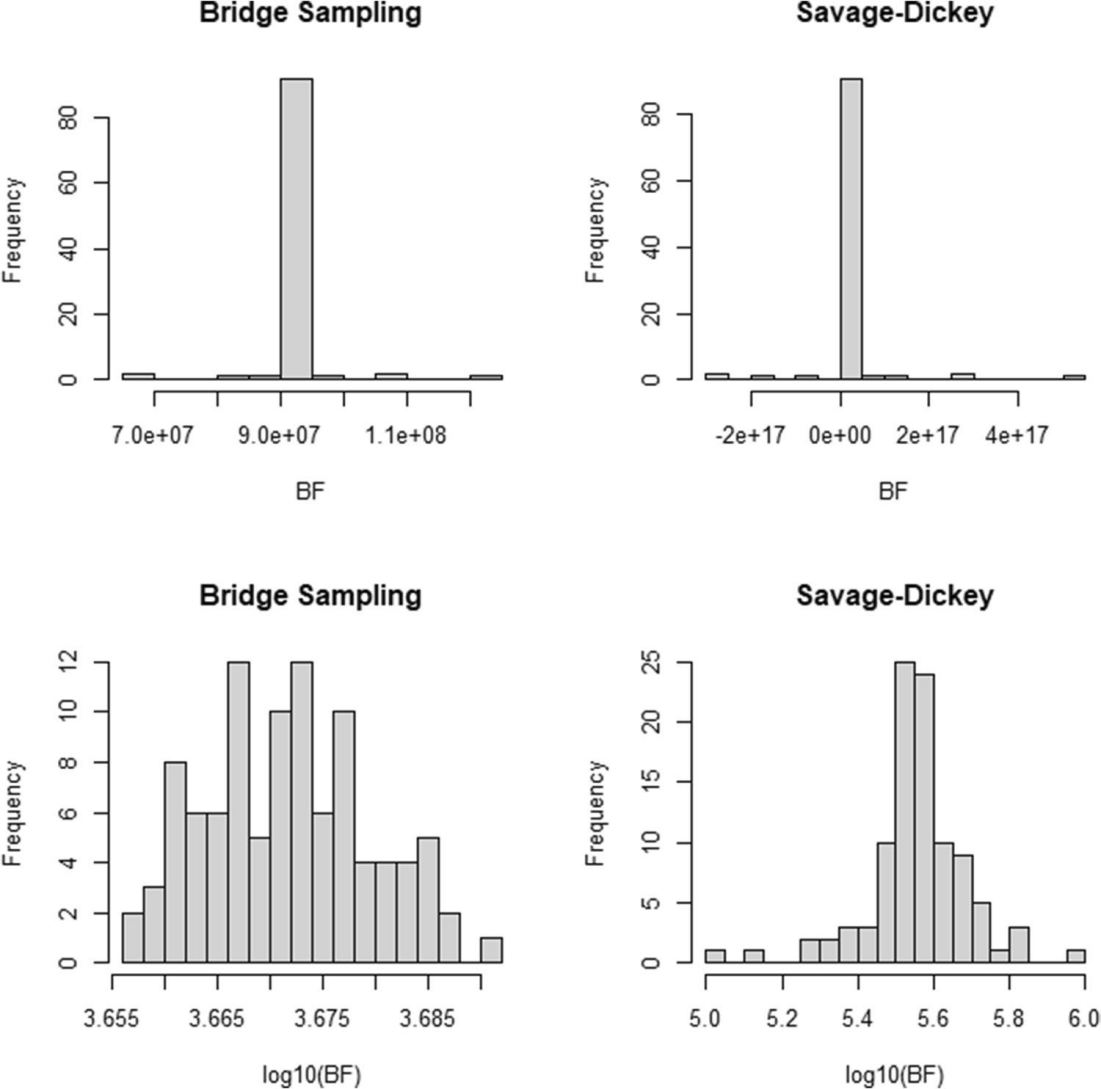
Distributions of Bayes factors (*top panels*) and log_10_(BF) (*bottom panels*) from Simulation 1a, 72,000 samples), for the main effect of IV 1. To remove variance between data samples, we subtracted the mean within each run from all BF estimates and added the grand mean across all runs at the end. This resulted in some negative BF values for the Savage–Dickey method because some runs had much larger means than the grand mean

To gauge the variance of BF estimates when we repeat the full data analysis on the same data, we repeated the procedure—sampling the posterior distribution and estimating the BFs—ten times for each simulated data set. For all analyses, we used logarithm of basis 10 of the BF_10_ (log_10_-BF_10_) as the variable of interest because raw BF_10_ values grow to astronomical numbers with larger effect and sample sizes, and their distributions are substantially skewed. The log-transformation results in approximately normal distributions (see Fig. 1 for example distributions).

Table 2 provides a summary of the results: For each level of the number of MCMC samples, the table shows the mean log_10_-BF across the ten simulations and the ten model-fitting runs per simulation. Positive log_10_-BF values reflect evidence in favor of an effect; negative values show evidence against the effect. A log_10_-BF value > 1 implies a BF > 10, which is usually regarded as strong evidence (Jeffreys, 1961; Lee & Wagenmakers, 2014; Wasserman, 2000); a log_10_-BF > 2 implies a BF > 100, and so on. Table 2 also presents the average standard deviation of log_10_-BF *within* each simulation.^7^ The SD within each simulation reflects the variance of BF estimates across the ten repeated analyses of the same data and thereby informs about the reliability of the BF estimation. Because the standard deviations are calculated on the logarithmic scale, they express the variance of BF estimates as proportions of their means on the original scale. For instance, a standard deviation of log_10_-BF of 0.02 means that the BF estimates have a dispersion of about 2% of the mean BF value.

**Table 2:** Log_10_ Bayes factors from Simulation 1a and 1b

	Normal DV				Binomial DV			
	Mean		Standard deviation		Mean		Standard deviation	
Term	Bridge sampling	Savage–Dickey	Bridge sampling	Savage–Dickey	Bridge sampling	Savage–Dickey	Bridge sampling	Savage–Dickey
IV1-within								
32,000 Samples	3.671	5.584	0.013	0.178	4.232	6.062	0.009	0.138
72,000 Samples	3.672	5.559	0.008	0.138	4.233	6.038	0.005	0.095
152,000 Samples					4.233	6.017	0.004	0.060
IV2-between								
32,000 Samples	3.782	5.504	0.012	0.212	3.757	5.183	0.009	0.119
72,000 Samples	3.784	5.527	0.007	0.132	3.757	5.178	0.005	0.088
152,000 Samples					3.757	5.174	0.004	0.063
Interaction 1 x 2								
32,000 Samples	– 1.184	– 1.175	0.010	0.005	– 1.001	– 0.991	0.008	0.004
72,000 Samples	– 1.183	– 1.174	0.007	0.004	– 0.999	– 0.991	0.005	0.003
152,000 Samples					– 0.998	– 0.991	0.003	0.002

### Simulation 1b: Binary dependent variable

Often behavioral responses are measured on binary scales, such as whether a person gave a correct or incorrect response to a test question. In that case, the data distribution is binomial rather than normal, and the linear model must be related to the data through a non-linear, typically logistic, link function. Simulation 1b uses the same design as Simulation 1a, except that the dependent variable was generated by a logistic model:

$$\begin{array}{l}{y}_{ij}\sim Binom({\lambda}_{ij})\\ {\lambda}_{ij}=Logistic\left({m}_{i}+{d}_{1,i}{X}_{1,i,j}+{d}_{2}{X}_{2,i}\right)\end{array}$$

We modeled the data with a hierarchical generalized linear model using the brms package (Bürkner, 2025). The model formula was the same as described for Simulation 1a, but we used a binomial distribution family with a logit link function on the linear model term. For the fixed effects, we used a Cauchy prior with scale = 0.35 because it generates a distribution of effect sizes that, when transformed to the probability scale, is widely spread without a strong concentration of density at the extremes. We again simulated ten data sets and repeated the analytic procedure ten times for each data set. This time, we used 5000, 10,000, or 20,000 MCMC samples per chain. The first 1000 samples of each chain were used as HMC warmup and discarded. This results in 32,000, 72,000, or 152,000 samples from the posterior distribution. Table 2 shows the results.

What is the risk of drawing erroneous conclusions about a hypothesis due to the variability of BF estimates? To assess this risk, we applied a very lenient conclusion criterion to the BF estimates, concluding in favor of an effect when BF > 3, and in favor of the null hypothesis when BF < 1/3. For each simulation (i.e., each simulated data set), we calculated the proportion of agreement in these decisions across the ten model-fitting runs for each hypothesis, separately for the BF estimates from the bridge-sampling and the Savage–Dickey method. When individual BF estimates were in the ambiguous range (1/3 < BF < 3), they were omitted because they do not support any decision. We found that the agreement was 100% for all hypothesis tests.

### Discussion

Both simulations show similar patterns of results: For the BFs obtained from bridge sampling, the SD (on the logarithmic scale) was consistently lower than 0.1. We find this tolerable because it will hardly lead to erroneous conclusions. If the true BF is actually 10% higher or lower than the estimate, our assessment of the strength of evidence would not change substantially. For the Savage–Dickey estimates of BF, the SD for small BFs that support the null hypothesis—as in the case of the interaction—were even narrower than for the bridge sampling BFs. By contrast, the SDs were much larger for large BFs, as obtained here for the two main effects. As these BFs were so large that they would unambiguously support the existence of the main effects even if they were an order of magnitude smaller, we could tolerate even this large degree of variability.^8^ A large degree of variability, however, would be problematic for BFs that provide weaker support for an effect. We designed Simulation 2 to explore BFs in favor of effects in a smaller range.

The variation of the number of MCMC samples had the expected effect: More samples resulted in more stable BF estimates. That said, even the smallest sample size (i.e., 32,000) was sufficient to obtain reasonably stable estimates in these simulations. The results for the normal and binomial data were comparable.

## Simulation 2: Varying effect sizes

In Simulation 2, we generated data from the same logistic model as for Simulation 1b. Here, we held the number of post-warmup MCMC samples constant at 72,000 (10,000 per chain, including 1000 warmup samples), and instead varied the effect size of IV 1 and IV 2 (0.1, 0.15, 0.2, 0.25). The effect sizes for both IVs were always the same. Table 3 shows the results. We again calculated the agreement proportion for decisions and found it to be 100%.

**Table 3:** Log_10_ Bayes factors from Simulation 2

	Mean		SD	
Parameters	Bridge sampling	Savage–Dickey	Bridge sampling	Savage–Dickey
IV1-Within				
Effect *d* = 0.1	– 0.036	– 0.016	0.006	0.022
Effect *d* = 0.15	0.715	0.933	0.005	0.038
Effect *d* = 0.2	1.275	1.515	0.005	0.035
Effect *d* = 0.25	2.767	3.586	0.005	0.07
IV2-Between				
Effect *d* = 0.1	– 0.31	– 0.319	0.005	0.013
Effect *d* = 0.15	1.549	1.835	0.005	0.053
Effect d = 0.2	2.388	3.157	0.005	0.058
Effect *d* = 0.25	3.463	4.643	0.005	0.066
Interaction 1 x 2				
Effect *d* = 0.1	– 0.985	– 0.978	0.005	0.003
Effect *d* = 0.15	– 0.973	– 0.965	0.005	0.003
Effect *d* = 0.2	– 1.02	– 1.012	0.005	0.003
Effect *d* = 0.25	– 0.94	– 0.933	0.004	0.003

The variability of BF from bridge sampling was again tolerably low throughout. This was the case regardless of the effect size. For the Savage–Dickey density ratio, the variability was larger whenever there was a true effect—as for the two main effects in the present simulation—but somewhat smaller when there was none—as for the interaction. Still, the standard deviations of the Savage–Dickey estimates are far from alarming. The BF values on the original scale increase with the size of the effect, and, as a result, the variability of BF estimates increases. However, the ratio of variability to the size of the BF values, which is reflected in the standard deviations on the logarithmic scale, remain approximately constant. In other words, when the Bayes factors are small, and estimation uncertainty could potentially lead to incorrect conclusions, the variability is low.

## Simulation 3: Increasing model complexity

In Simulation 3, we explored whether the variability of BF estimates increases with more complex models than those we studied so far. We added a third IV to the experimental design, which had two levels varying within subjects. This substantially increases the number of model parameters.^9^ The data-generating process included linear main effects of all three IVs (with effect sizes *d*_1_ = 0.25, *d*_2_ = 0.25, and *d*_3_ = 0.05) and an interaction between IVs 1 and 3 (*d*_13_ = 0.25). We reduced the number of trials per design cell to 50 so that the overall amount of data is still in the typical range for behavioral lab experiments. To reduce computational costs, in Simulations 3 and 4, we followed a different model-comparison strategy for estimating BFs through bridge sampling than in the preceding simulations. We tested each effect by comparing models that omitted all higher-order effects. For example, to test the main effect of IV1, we compared a reference model including main effects for all IVs (but no interactions) to a restricted model that additionally omitted the main effect of IV1. These model comparisons adhere to the principle of marginality.^10^

We ran this simulation in two versions, one using a linear model with a normal distribution for data generation and as a statistical model (Simulation 3a; aggregating data within design cells to accelerate computation), and one using a logistic model with a binomial distribution (Simulation 3b). The model was applied to the data with 32,000, 72,000, and 152,000 MCMC samples. Table 4 summarizes the results.

**Table 4:** Log_10_ Bayes factors from Simulation 3a and 3b

	Simulation 3a: Normal DV				Simulation 3b: Binomial DV			
	Mean		Standard deviation		Mean		Standard deviation	
Term	Bridge sampling	Savage–Dickey	Bridge sampling	Savage–Dickey	Bridge sampling	Savage–Dickey	Bridge sampling	Savage–Dickey
IV1-Within								
32,000 Samples	3.936	3.152	0.010	0.091	2.472	6.970	0.010	0.140
72,000 Samples	3.936	3.153	0.006	0.051	2.472	6.981	0.007	0.093
152,000 Samples	3.935	3.164	0.003	0.038	2.473	6.969	0.004	0.067
IV2-Between								
32,000 Samples	3.198	5.077	0.010	0.095	3.648	3.921	0.011	0.10
72,000 Samples	3.197	5.072	0.005	0.07	3.65	3.916	0.005	0.058
152,000 Samples	3.197	5.086	0.003	0.038	3.649	3.920	0.004	0.038
IV3-Within								
32,000 Samples	– 0.393	– 0.552	0.009	0.013	– 0.192	0.130	0.010	0.025
72,000 Samples	– 0.393	– 0.553	0.006	0.009	– 0.191	0.133	0.006	0.016
152,000 Samples	– 0.393	– 0.552	0.004	0.004	– 0.192	0.132	0.004	0.011
Interaction 1 x 2								
32,000 Samples	– 1.198	– 1.123	0.010	0.003	– 0.943	– 0.982	0.011	0.003
72,000 Samples	– 1.198	– 1.123	0.006	0.002	– 0.943	– 0.982	0.006	0.003
152,000 Samples	– 1.198	– 1.123	0.004	0.001	– 0.943	– 0.982	0.004	0.002
Interaction 1 x 3								
32,000 Samples	31.943	32.999	0.009	0.408	40.287	35.811	0.010	0.416
72,000 Samples	31.944	32.942	0.006	0.322	40.288	35.877	0.006	0.325
152,000 Samples	31.944	32.970	0.004	0.205	40.288	35.835	0.004	0.198
Interaction 2 x 3								
32,000 Samples	– 1.092	– 1.109	0.011	0.004	– 0.930	– 0.940	0.010	0.004
72,000 Samples	– 1.091	– 1.109	0.005	0.002	– 0.930	– 0.940	0.006	0.002
152,000 Samples	– 1.091	– 1.109	0.004	0.002	– 0.93	– 0.939	0.004	0.002
3-Way Interaction								
32,000 Samples	– 1.517	– 1.531	0.010	0.003	– 1.365	– 1.263	0.009	0.004
72,000 Samples	– 1.517	– 1.531	0.006	0.001	– 1.366	– 1.262	0.006	0.002
152,000 Samples	– 1.516	– 1.531	0.004	0.001	– 1.367	– 1.263	0.004	0.002

For the more complex experimental design explored here, the SD estimates were comparable to those of the simpler designs in the preceding simulations. Again, the variability of BF estimates from bridge sampling was tolerable throughout. For BFs in support of an existing effect, the variability of Savage–Dickey estimates was substantially larger than that from bridge sampling, but never anywhere near as large as to risk drawing incorrect conclusions. Therefore, we consider this level of variability tolerable. This is supported by the observation that the proportion of decision agreements was again 100%. Once again, increasing the number of MCMC samples had the expected effect of reducing the variability of BF estimates across both estimation methods.

For the non-zero effects—the main effects of IV1 and IV2, and the IV1 x IV3 interaction—the Savage–Dickey density ratio not only led to more variable BF estimates than the bridge sampler but also to larger BFs on average. This was also observed in all previous simulations and seems to reflect a general tendency of Savage–Dickey BF estimates to be overestimated when the true BF is large.

## Simulation 4: Increasing the number of parameters

For this simulation, we used the relatively complex design of Simulation 3 and increased the number of to-be-estimated parameters further by simulating data from a larger subject sample (N_subj_ = 100 per experimental group). This increases the dimensionality of the parameter space for which the MCMC sampler needs to explore the likelihood by a factor of about 5, and thereby challenges the estimation of BFs even more than in the preceding simulations.^11^ We ran only the logistic model because the computation time for the linear model is substantially longer. To save computation time, we ran only simulations with 5000 samples per chain (retaining 32,000 samples across eight chains), the smallest number investigated in previous simulations, which should be most diagnostic for estimation problems. We set the effect sizes of all the true effects—the three main effects and the interaction of IV 1 with IV 3—to *d* = 0.05 with the aim to obtain BFs closer to the ambiguous range, for which precise BF estimates are particularly important. We created 23 simulated data sets, each of which was analyzed ten times. Table 5 shows the results.

**Table 5:** Log_10_ Bayes factors from Simulation 4

	Mean		Standard deviation	
Term	Bridge sampling	Savage–Dickey	Bridge sampling	Savage–Dickey
IV1-Within	0.011	0	0.348	0.027
IV2-Between	– 0.404	– 0.403	0.34	0.02
IV3-Within	0.287	0.299	0.364	0.036
Interaction 1 x 2	– 1.322	– 1.311	0.428	0.004
Interaction 1 x 3	0.182	0.246	0.43	0.034
Interaction 2 x 3	– 1.328	– 1.298	0.37	0.004
3-way interaction	– 1.399	– 1.304	0.486	0.004

Compared to Simulation 3b, the results of Simulation 4 with bridge sampling are much more variable. The BF estimates from the Savage–Dickey estimation were more robust against the increase in subject sample size. Their variability was, if anything, even smaller than in Simulation 3b. When the BF estimates are used as the basis for a decision about a hypothesis, the variability of the bridge-sampling BF estimates in Simulation 4 can influence such decisions: For four effects (the three main effects and the interaction of IV1 and IV3), we found agreement proportions between 90 and 100%. For the Savage–Dickey based BF estimations, the decision agreement was 100% throughout.

The opposite effects of the changes we made between Simulations 3b and 4 on the two BF estimation methods show how their variances are sensitive to different simulation settings: The estimates from bridge sampling are *less* reliable in Simulation 4 because of the increased number of free parameters that the larger number of subjects entails. The estimates from the Savage–Dickey method are *more* reliable because the data-generating model of Simulation 4 included only small effects and only modest evidence for the presence of effects.

Compared to Simulation 3b, the average Savage–Dickey estimates for BFs reflecting true effects are considerably smaller and in better agreement with the bridge sampling estimates. This is likely the result of two differences between these simulations. One is that the true effects were smaller in Simulation 4, yielding smaller BFs by both estimation methods. This implies that zero—the point at which the posterior density is evaluated—never lies in the far tails of the posterior distributions, at which their density is difficult to estimate. We explore this point further in Simulation 5. The other difference between simulations 3b and 4 is that we increased the sample size compared to the previous simulation. According to Bayesian central limit theorem (Bernardo & Smith, 2000), in parametric models, the multivariate posterior distributions will asymptotically be normally distributed. Hence, increasing the sample size will improve the normal approximation to the marginal posterior distribution and reduce potential bias in the estimation of the BF (cf., Morey et al. 2011)—in addition to increasing model complexity. This suggests that the average Savage–Dickey estimates in previous simulations were larger than bridge sampling estimates because the marginal posterior distributions deviated from normality. We explore these results further in Simulation 5.

## Discussion of Simulations 1–4

Simulations 1–4 have produced several results that will be useful to the practical researcher trying to decide what BF estimation method to choose and how to use it. First, the variability of Savage–Dickey density-ratio estimates increased with the strength of the evidence for an effect. In contrast, the variability of bridge sampling estimates appeared to be constant across a wide range of BF. This result is evident across all simulations, but is clearest in Simulation 2. As a result, Savage–Dickey estimates were more variable than the bridge sampling estimates when there was strong evidence for an effect (Simulations 1, 2, and 3) but comparable (Simulations 1–4) and sometimes less variable when there was weak evidence for, or clear evidence against an effect (Simulations 3 and 4). Critically, the variance of Savage–Dickey estimates was relatively small when the evidence in favor of an effect was modest. Hence, there was little risk of drawing incorrect conclusions due to the variance of the estimates.

As expected, the variance of both estimation methods decreased when we increased the MCMC sample size (Simulations 1 and 3), but even modest sample sizes (32,000) yielded surprisingly stable estimates (Simulations 1, 3, and 4). In most simulations, we found that the Savage–Dickey density ratios yielded larger BF estimates than bridge sampling when the evidence for an effect was strong (Simulation 1, 2, and 3), but comparable estimates when there was evidence against an effect (Simulations 1–4). Our findings do not seem to depend on the distribution of the dependent variable or the link function of the hierarchical model (Simulations 1 and 3).

Simulation 4 produced two additional results that warrant further investigation. We observed that increasing the number of participants had two effects: The average BF estimates of bridge sampling and of Savage–Dickey density ratio were in better agreement, even when the variability of bridge sampling estimates was increased compared to Simulation 3. We designed Simulation 5 and 6 to better understand these results. We suspect that the average Savage–Dickey estimates in previous simulations were larger than bridge sampling estimates because the marginal posterior distributions deviated from normality and hence yielded biased posterior density estimates. In practice, the risk of biased estimates can be mitigated by visually inspecting the fit of the estimated probability density function to the posterior samples or by using a less assumptive density estimation approach. However, as shown by Morey et al. (2011), the resulting estimates can be more variable and may still be biased when the evidence for an effect is large. We explored the relationship between bias and variance for three commonly used density estimators in Simulation 5.

## Simulation 5: Density approximation methods for the Savage-Dickey ratio

In all previous simulations, we approximated the posterior density at the test value using a normal distribution (Bernardo & Smith, 2000). General-purpose implementations of the Savage–Dickey density ratio in the R packages bayestestr (Makowski et al., 2019) and brms^12^ (Bürkner, 2025) use more flexible approximations—logspline density (LS) estimates and spline-smoothed kernel (SSK) density estimates (Wetzels et al.,  2009). If the posterior distribution is decidedly non-normal, a normal approximation can result in biased BF estimates. The logspline and spline-smoothed kernel density estimates are more flexible to approximate non-normal posterior distributions and can reduce bias in BF estimates. This flexibility, however, comes at the cost of higher variance (i.e., lower reliability). What is more, Morey et al. (2011) report that in approximately normal posterior distributions, the logspline density method can be *more* biased than the normal approximation—in particular when ${\mathrm{BF}}_{10}$ is large. This is because the more flexible methods must estimate the shape of the tails of the distribution where the number of MCMC samples decreases rapidly and simplifying assumption must be made (e.g., Kooperberg & Stone, 1991). Hence, in Simulation 5, we compared bias and reliability of the normal approximation to logspline density (LS) and the spline-smoothed kernel (SSK) density estimates at varying tail probabilities of the posterior distribution and MCMC samples sizes.

In our previous simulations, we have explored the number of MCMC samples necessary to obtain reliable BF estimates. Owing to the computational resources required by the bridge sampling algorithm, we varied the MCMC sample size in only three steps and across a limited range (32,000–152,000). By focusing on the Savage–Dickey density ratio, in Simulation 5 we were able to examine the effect of the MCMC sample size on the bias and reliability of BF estimates more closely. We varied the MCMC sample size across a larger range (500–500,000) and in ten steps.

**Fig. 2:**
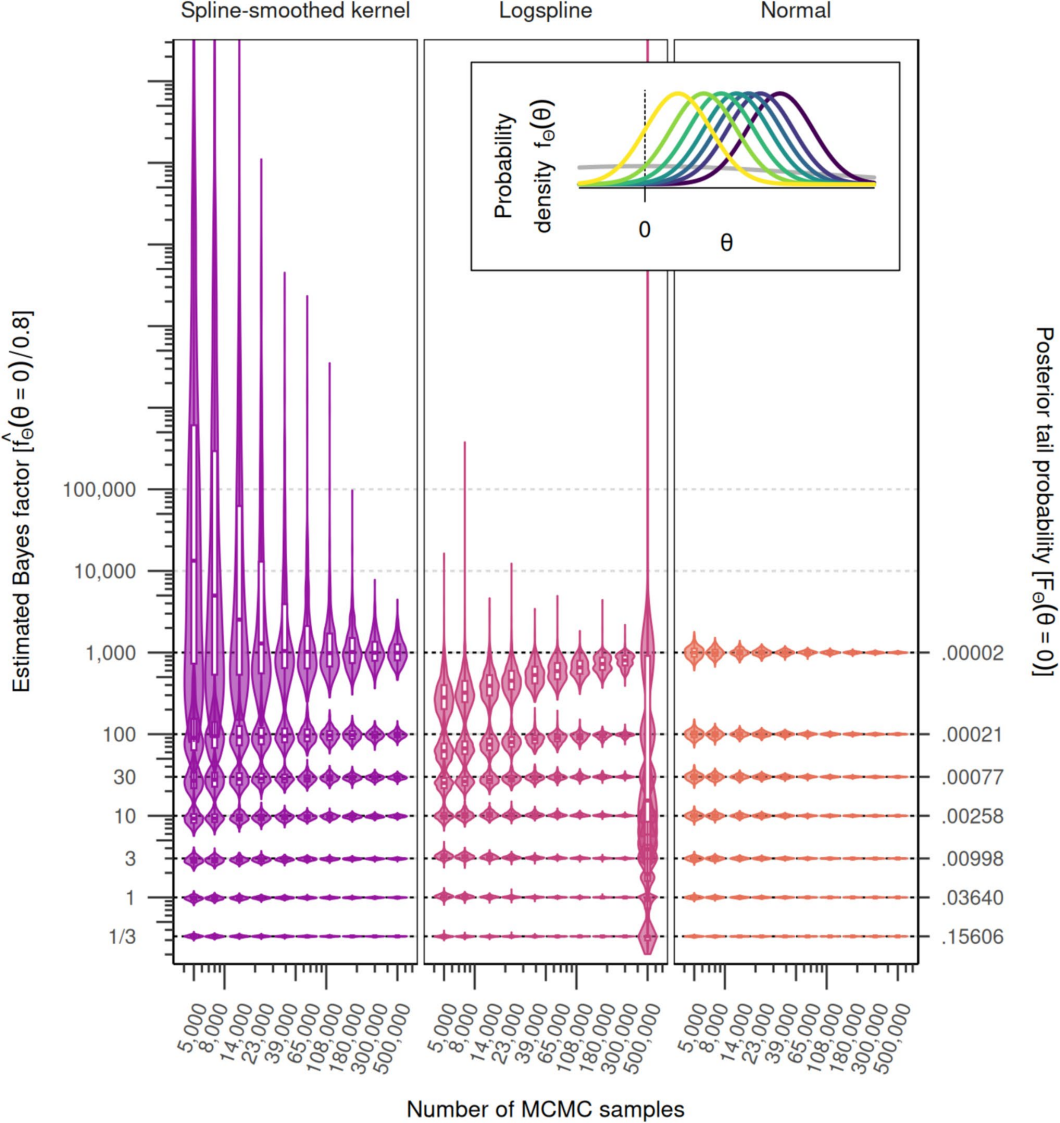
Bias and variability of estimated probability densities $\widehat{{f}_{\Theta }}\left(\theta =0\right)$  as a function of MCMC sample size and three posterior density estimation methods: Spline-smoothed kernel density, logspline density, and best-fitting normal probability density. Probability densities were estimated for seven normal distributions $N\left(\mu ;\sigma =1\right)$ with cumulative probability densities ${F}_{\Theta }\left(\theta =0;\mu , \sigma \right)$, where μ was chosen to yield typical BF category borders when assuming a prior distribution implying a reasonable amount of belief updating ($N\left(\mu =0;\sigma =5\right)$). The *grey curve* in the inset represents the prior distribution; the *colored curves* represent the posterior distributions with shifting means. *Violins* and *boxplots* show the distributions of estimates for each MCMC sample size (labeled on the *X*-axis), and each true probability density (*horizontal lines*). *Axes* show log_10_-scale, i.e., orders of magnitude

The potential for bias in the normal approximation to the posterior distribution is arbitrarily large as it is a direct consequence of the discrepancy in the shape of the posterior distribution. It is easy to create conditions under which this method performs poorly. Our goal for this simulation was to further gauge the performance of the more flexible methods and use the normal approximation as a benchmark. We therefore simulated posterior samples from normal distributions, $N\left(\mu ;\sigma =1\right)$. As we expected the differences between estimation methods to be particularly noticeable in the tails of the distribution, we varied the mean μ of the posterior distribution from 0 to increasing positive values, corresponding to increasingly larger true effects and smaller tail probabilities at the test value 0 (i.e., smaller cumulative probability densities for $\theta \le 0$). To put the estimated posterior densities $\widehat{{f}_{X}}\left(x=0\right)$ on a scale that is more readily interpretable and consistent with previous simulations, we report Savage–Dickey density ratios assuming a prior density that implies a fair amount of updating: We assumed a rather uninformative normal prior with μ = 0 and σ = 5. To compute the Savage–Dickey density ratio, we divide the prior density at 0 by the posterior density at 0. Thereby, the density of the prior at 0, ${f}_{X}\left(\mathrm{x}=0; \upmu =0, \upsigma =5\right)=0.798$, effectively serves as a scaling constant for the inverse posterior density at 0. Since we chose the prior arbitrarily to scale the posterior densities, the absolute magnitudes of the Bayes factors in Fig. 2 are also arbitrary. We chose posterior means that yield BFs that correspond to boundaries of intervals from popular interpretation guidelines, that is, ${\mathrm{BF}}_{10}\in \left[{~}^{1}\!\left/ \!{~}_{3}\right., 1, 3, 10, 30, 100, 1000\right]$. We estimated 1000 probability densities in each condition of the simulation.

Before we discuss the results, we reiterate that in all simulations Savage–Dickey density ratios were estimated using the analytic prior density, and the posterior density was estimated using MCMC samples from the posterior. In some applications, however, the prior density may not be available analytically, for example, because the test-relevant parameter is calculated from other model parameters. In such instances, the density estimation procedures must also be used to estimate the prior density from MCMC samples. This approach is the default in some general-purpose implementations of the Savage–Dickey density ratio (e.g., Bürkner, 2025; Makowski et al., 2019). The resulting estimator of the BF compounds uncertainty about the prior and posterior densities and will likely be less reliable than our results suggest. Although most of the prior density is typically centered around 0 and estimation of the prior density at 0 should be achievable with relatively high precision, we recommend using the analytical prior whenever available.

### Results

The normal approximation of the posterior distributions produced unbiased and reliable density estimates even for modest MCMC sample sizes, Fig. 2. As expected, the variability of the estimates increased as the posterior tail probability of the test value ${F}_{\Theta }\left(\theta =0;\mu , \sigma =1\right)$ decreased. Yet, even for our smallest MCMC sample size of 5000 the density estimates were so reliable that all interpretation categories on the BF scale could be distinguished unambiguously. As the MCMC sample size approached 40,000 there was no noteworthy uncertainty in the estimated BFs.

As expected, the more flexible density estimation methods produced more variable but also more biased estimates. In both methods, the variance of estimates increased as the posterior tail probability decreased (i.e., the posterior moved farther away from 0) and decreased as MCMC sample size increased. The pattern of bias was more complex. The logspline approximation exhibited an upward bias for moderate posterior tail probabilities ${F}_{\Theta }\left(\theta =0\right)>.010$, which was negligible across all MCMC sample sizes. For small posterior tail probabilities ${F}_{\Theta }\left(\theta =0\right)<.003$, estimates were biased downwards. When the number of MCMC samples was small, this bias and the variability of the estimates made it difficult to distinguish between BF interpretation categories. For the most extreme posterior tail probability, a noticeable downward bias persisted even for 300,000 MCMC samples. In our view, bias and reliability of the estimates reached acceptable levels at an MCMC sample size of approximately 40,000 in all conditions. If feasible, we recommend samples sizes of 65,000 or more.

For 500,000 MCMC samples, the logspline estimation procedure failed catastrophically. In two simulation runs, logspline density estimates were negative or infinite, and across all runs, the estimates were strongly biased and highly variable. In our experience, it is unusual to draw as many as 500,000 posterior samples. Hence, we do not consider this algorithmic failure a fatal flaw of the method—it performed well for more typical sample sizes. Yet, users of the logspline method should be wary of such algorithmic failures when using many MCMC samples.

The spline-smoothed kernel approximation exhibited a downward bias for moderate to small posterior tail probabilities ${F}_{\Theta }\left(\theta =0\right)>.001$, which was more pronounced than in the logspline method but relatively small across all MCMC sample sizes. For very small posterior tail probabilities ${F}_{\Theta }\left(\theta =0\right)<.0002$, estimates were increasingly biased upwards. Compared to logspline estimates, the bias decreased more quickly with the number of MCMC samples.^13^ Across all conditions, the spline-smoothed kernel estimates were more variable than the logspline estimates.

When the number of MCMC samples was small, this bias and variability made distinguishing between BF interpretation categories difficult. In our view, bias and variability of the estimates reached acceptable levels at an MCMC sample size of 65,000 for all conditions. If feasible, we recommend sample sizes of approximately 100,000 or more—approximately 50% more than for the logspline approximation.

For moderate posterior tail probabilities ${F}_{\Theta }\left(\theta =0\right)>.01$, all methods produced relatively reliable estimates with no or negligible bias—even for MCMC sample sizes as small as 5000–10,000.

### Discussion

**Fig. 3:**
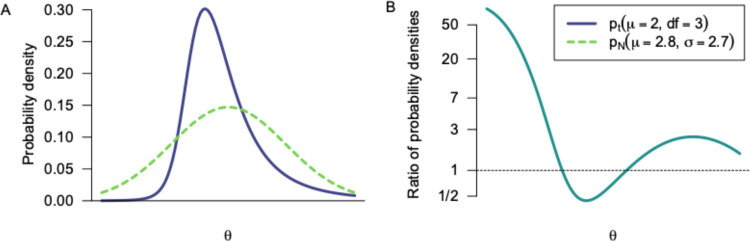
Illustration of bias resulting from inappropriately approximating a posterior distribution with a normal distribution. **A** Probability density distributions of a non-central *t* distribution $t\left(\mu =2, \nu =3\right)$ and the normal distribution with the same mean and variance. **B** Factor (on log-scale) by which the probability density of the normal approximation overestimates the true probability density

Our results highlight how parametric assumptions about the posterior distribution—here, the assumption that it is approximately normally distributed—can yield very efficient estimates of posterior densities. This high reliability comes with the risk of a potentially large bias when applied blindly, even though the parametric assumption is inappropriate. Consider the example of a posterior distribution that follows a non-central *t* distribution $t\left(\mu =2, \nu =3\right)$—skewed and heavy-tailed compared to a normal distribution. Figure 3 shows that the normal approximation can underestimate the true probability density by a factor of 2.5 or severely overestimate the probability density by a factor of 20 or more. In such situations, more flexible density approximations promise to be less biased at the cost of being more variable. However, our simulation shows that these methods can be very biased when the to-be-estimated density is deep in the tails of the posterior distribution, and the number of MCMC samples is modest. In such cases, spline-smoothed kernel estimates are substantially more variable than logspline estimates, but they can be less biased than logspline estimates when the number of MCMC samples is large. Overall, our results suggest that logspline estimates may be a better default for general-purpose implementations of the Savage–Dickey ratio, because the estimates are more reliable and what bias occurs in the tails of the distribution appears to bias the estimates towards lower BFs—that is, it errs on the side of under- rather than overstating the evidence. However, firm conclusions require additional simulations for a wider range of posterior distributions. We leave these simulations for future research.

## Simulation 6: Varying strength of evidence and model complexity

The results of Simulations 2, 3, and 4 suggest that the variance of BF estimates obtained by bridge sampling and by the Savage–Dickey density ratio may be differently affected by the magnitude of the true BF and the number of model parameters: Whereas the Savage–Dickey density ratio is unaffected by the number of model parameters, its variance increases with the evidence for an effect—that is, as the posterior tail probability of the test value ${F}_{\Theta }\left(\theta =0\right)$ decreases. Conversely, the variability of bridge sampling estimates increases with the number of model parameters, but it is unaffected by the magnitude of the true BF. To confirm these results, we conducted a final simulation, in which we varied these factors directly and independently.

In this simulation, we added one more method for computing the Savage–Dickey density ratio: conditional marginal density estimation (CMDE). This method requires that the conditional posterior distribution $p\left(\theta \right|\phi , {\boldsymbol{y}}, {\mathcal{M}}_{1})$ is known. When this is the case, the estimator can be efficiently sampled during the MCMC simulation and, due to Rao-Blackwellization, is very reliable. It can be considered a best-case scenario for the Savage–Dickey density ratio. Morey et al. (2011) showed that this method is substantially more reliable and less biased than the logspline and normal approximations of the posterior probability density. However, note that the conditional posterior distribution differs across models and is often unknown for even moderately complex models, such as hierarchical generalized linear models. Hence, it is not one of the generally applicable, easy-to-use methods explored in our previous simulations. We include it here for comparison.

We also explored whether the reliability of bridge-sampling estimates from the bridge-sampling R package (Gronau et al., 2018) differs across parameter types. Specifically, we tested whether the estimation may be less reliable for models with large correlation matrices, such as hierarchical models with more random effects. We therefore compared the effects of the number of independent, unbounded continuous parameters (e.g., independent means) and of a single large Cholesky-factorized correlation matrix (p. 273, Stan-Development-Team, 2024).

For this simulation, we manipulated the true BF by simulating data for a one-sample *t* test, for which the true BF is available analytically. We simulated six datasets, consisting of 100 observations each, that yield Bayes factors of ${\mathrm{BF}}_{10}\in \left[{~}^{1}\!\left/ \!{~}_{9}\right.,{~}^{1}\!\left/ \!{~}_{3}\right., 1, 3, 10, 30, 100\right]$ in default JZS *t* tests (Rouder et al., 2009). The unconstrained model has two parameters: the mean and the standard deviation of the data-generating normal distribution. To explore the effect of the number of model parameters on the BF estimation methods, we introduced additional parameters that contributed nothing to the model predictions; that is, they did not affect the models’ marginal likelihood. These parameters were unrelated to the data that was the subject of the *t* test, were thus irrelevant to the BF, and were not updated by data—they were sampled from their assigned prior distributions. To achieve this artificial inflation of the dimensionality of the joint parameter distribution, we implemented the null and alternative models in the probabilistic programming language Stan (Stan-Development-Team, 2024) and added sampling statements for parameters that did not factor in the models’ likelihood. Specifically, we sampled values for 3, 435, or 780 parameters from either independent standard normal distributions or a Cholesky-factorized LKJ distribution with η=2 degrees of freedom. The purpose of these irrelevant parameters was to increase the number of model parameters, while holding all other variables constant, such as the true BF, the data informing the model comparison, or the number of true effects in the data-generating process.

To keep this simulation at a manageable scale, we opted to use a fixed number of MCMC samples. The results of Simulation 5 suggest that 100,000 samples yield relatively unbiased and stable estimates for all density estimation methods. Following 2000 warm-up samples, we drew 102,000 posterior samples across six chains. We repeated this procedure 30 times for each dataset. We monitored the sampler for divergent transitions, as well as the Bayesian fraction of missing information, whether the sampling algorithm reached the maximum tree depth, and the maximum $\widehat{R}$ across all parameters. According to these diagnostics, the sampler converged to the stationary distribution and explored the joint posterior efficiently.^14^

Different from Simulations 1–4, we used the Warp-III method of the bridge sampler. One attractive property of Warp-III bridge sampling is that it makes more lenient assumptions about the shape of the full posterior distribution. Warp-III bridge sampling is, therefore, an alternative to spline-smoothed kernel and logspline density estimation when the posterior distribution is non-normal. The Warp-III bridge sampling algorithm converged within 3–9 iterations.^15^ We repeatedly estimated the marginal likelihood 30 times for each of the 30 sets of posterior samples. We compared bias and variance of individual estimates to the average of the 30 repeated estimates.

### Results

**Fig. 4:**
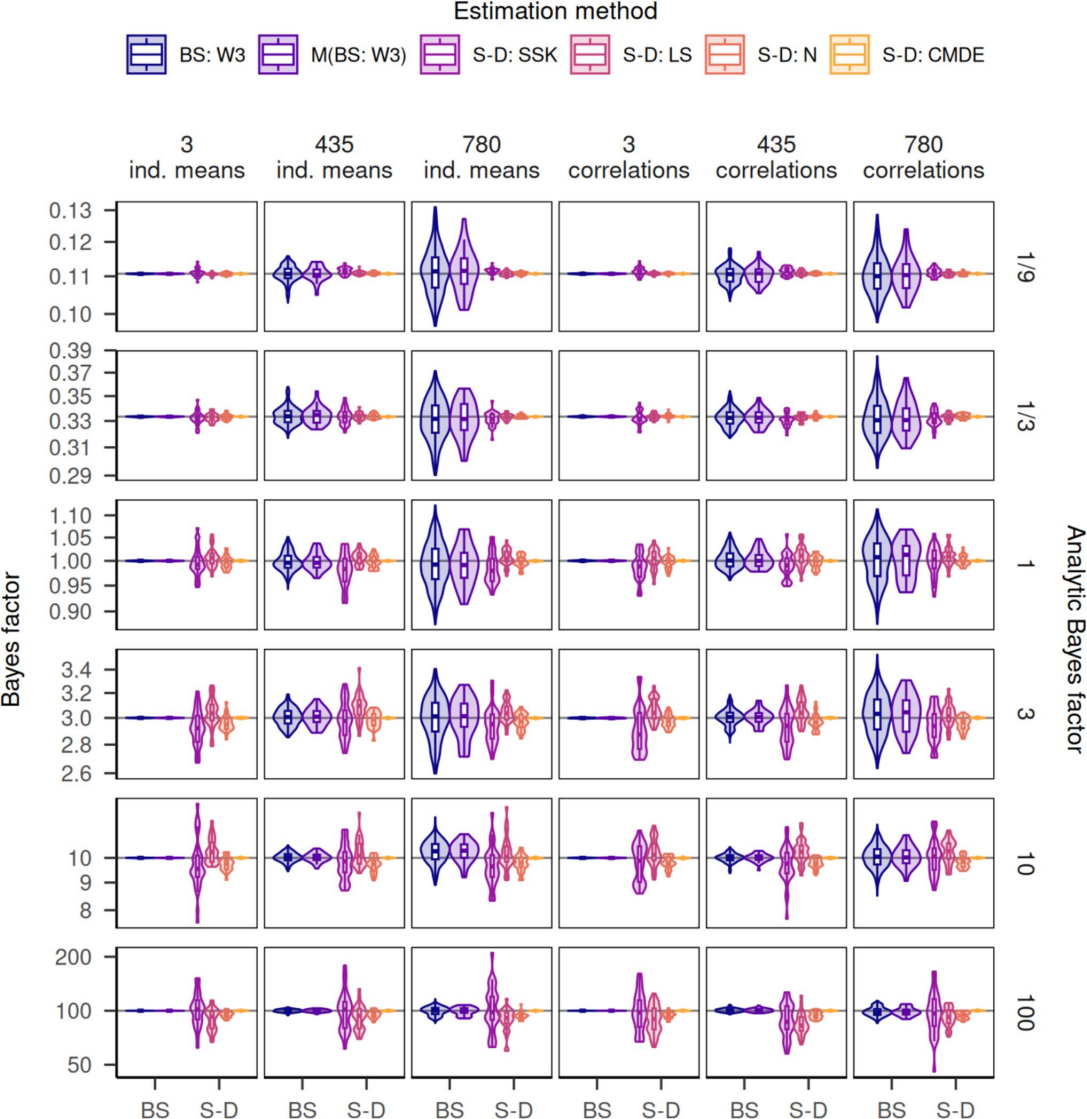
Bayes factors estimated using the Warp-III bridge sampling algorithm in two variants (BS: W3 = 30 individual estimates for each of 30 sets of posterior samples; M(BS: W3) = means across the 30 individual estimates for each of 30 sets) or Savage–Dickey density ratio (S-D) based on four different posterior density estimates: Spline-smoothed kernel density (SSK), logspline density (LS), normal probability density (N), and conditional marginal density estimation (CMDE). Bayes factors were estimated for six datasets with analytic BF varying from 1/9 to 100. To vary the dimensionality of the posterior, we added 3, 435, or 780 parameters that were unrelated to the model likelihood. Violins and boxplots show the distributions of Bayes factor estimates on log_10_-scale, i.e., orders of magnitude

**Fig. 5:**
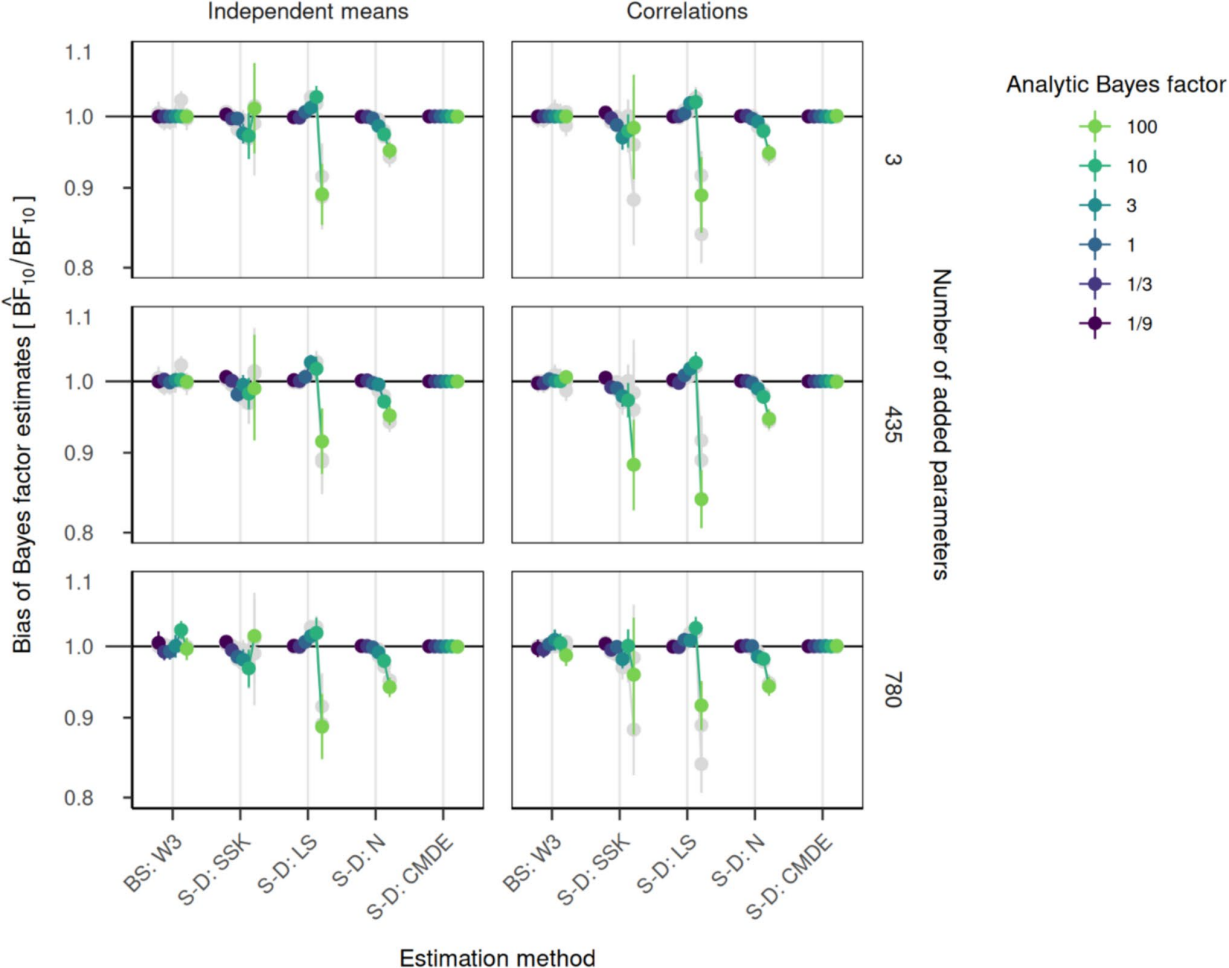
Proportional bias of Bayes factors estimated using the Warp-III bridge sampling algorithm (BS: W3) or Savage–Dickey density ratio (S-D) based on four different posterior density estimates: Spline-smoothed kernel density (SSK), logspline density (LS), normal probability density (N), and conditional marginal density estimation (CMDE). Bayes factors were estimated for six datasets with analytic BF varying from 1/9 to 100. To vary the dimensionality of the posterior, we added 3, 435, or 780 parameters that were unrelated to the model likelihood. *Points* represent posterior medians, and *error bars* 95% credible intervals. *Grey points* show results for different numbers of added parameters for comparison

**Fig. 6:**
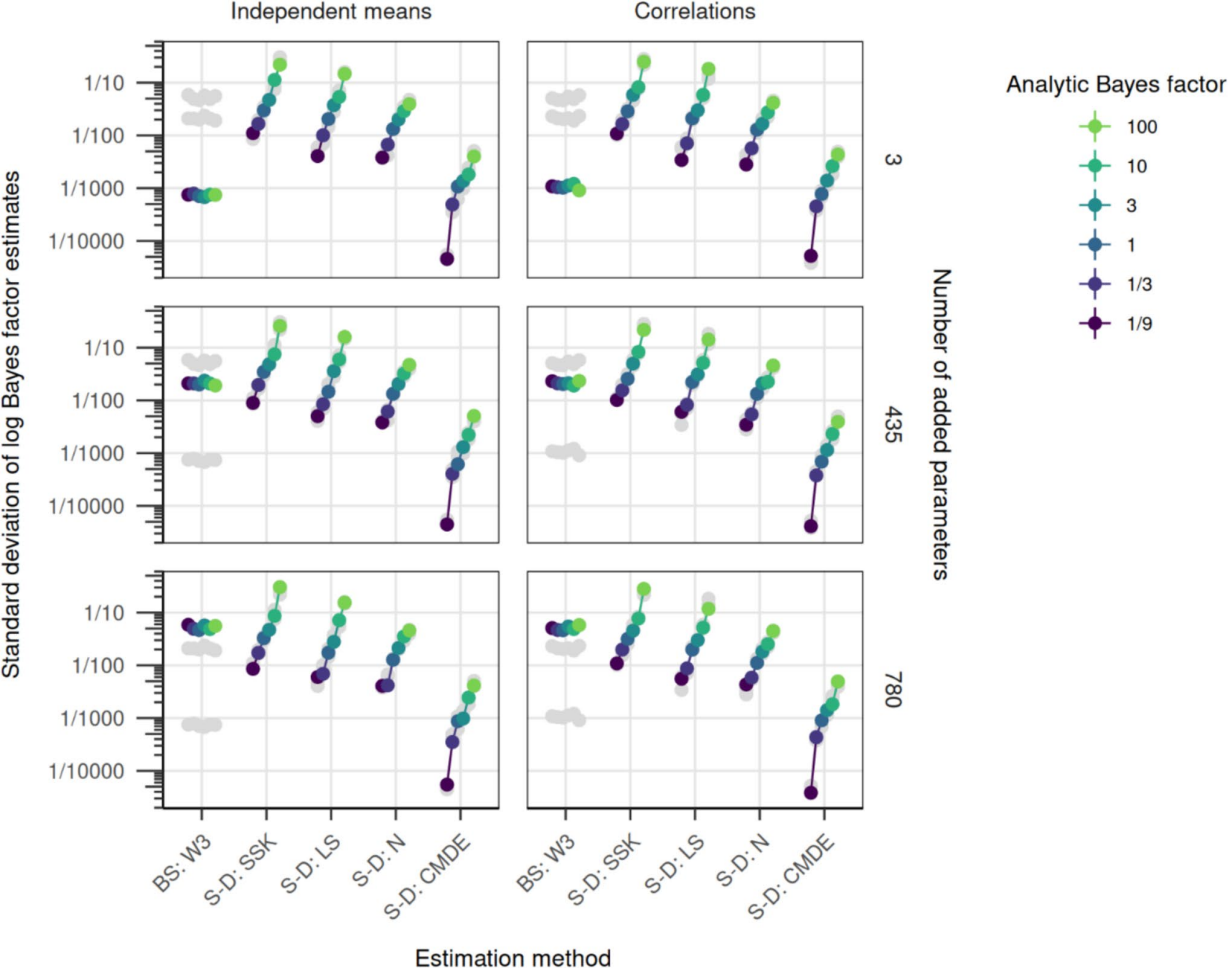
Standard deviations (on logarithmic scale) of log Bayes factors estimated using the Warp-III bridge sampling algorithm (BS: W3) or Savage–Dickey density ratio (S-D) based on four different posterior density estimates: Spline-smoothed kernel density (SSK), logspline density (LS), normal probability density (N), and conditional marginal density estimation (CMDE). Bayes factors were estimated for six datasets with analytic BF varying from 1/9 to 100. To vary the dimensionality of the posterior, we added 3, 435, or 780 parameters that were unrelated to the model likelihood. *Points* represent posterior medians and *error bars* 95% credible intervals. *Grey points* show results for different numbers of added parameters for comparison

Figure 4 shows the distributions of BF-estimates from our simulation. To explore bias and reliability (i.e., variance) separately, we estimated each method’s bias as the ratio of estimated and true Bayes factor (${\widehat{\mathrm{B}\mathrm{F}}}_{10}/{\mathrm{BF}}_{10}$), and each method’s variance as the standard deviation of estimated log Bayes factors, $\mathrm{S}\mathrm{D}\left\{\mathrm{log}\left({\widehat{\mathrm{B}\mathrm{F}}}_{10}\right)\right\}$ fitting separate (hierarchical) linear models to the estimated log Bayes factors of each condition using brms with default priors suitable for estimation. Figure 5 highlights each method’s bias; Fig. 6 highlights each method’s variance. Overall, the results confirm our conclusions from previous simulations. The different types of added parameters yielded consistent results.

#### Bias

As shown in Fig. 5, the results for the Savage–Dickey density ratios are generally in line with our findings from Simulation 5. With more than 100,000 samples from the posterior, the bias of all methods was mild. Logspline estimates exhibited the largest bias, underestimating the true Bayes factor of ${\mathrm{BF}}_{10}=100$ by approximately 10%.

Estimates from the normal approximation increasingly underestimated the true BF as the evidence for an effect increased. At a true ${\mathrm{BF}}_{10}=100$, bias was approximately 5%. As in Simulation 5, the pattern of biases was more complex for the more flexible density estimation methods. Logspline estimates were unbiased when ${\mathrm{BF}}_{10}<1$, slightly overestimated moderate Bayes factors of $1<{\mathrm{BF}}_{10}<10$, but underestimated the true ${\mathrm{BF}}_{10}=100$. In contrast, Spline-smoothed kernel density estimates slightly overestimated true ${\mathrm{BF}}_{10}=1/9$, but increasingly underestimated moderate Bayes factors of $1/3<{\mathrm{BF}}_{10}<10$. For true values of ${\mathrm{BF}}_{10}=100$ the results were ambiguous, and our bias estimates were uncertain. This is consistent with our results from Simulation 5, in which we saw a switch from under- to overestimation and substantial increases in the variability of estimates as the true evidence increased. As expected, conditional marginal density and bridge sampling estimates were unbiased in all conditions.

#### Reliability

As shown in Fig. 6, the results for the Savage–Dickey density ratios, again, were in line with our findings from Simulation 5. The simulation confirmed that the variability of the Savage–Dickey density ratios depended only on the size of the BF—that is, variability increased as the posterior tail probability of the test value ${F}_{\Theta }\left(\theta =0\right)$, and with it the true BF, increased. In contrast, the variability of bridge sampling only increased noticeably with the number of model parameters.

**Fig. 7:**
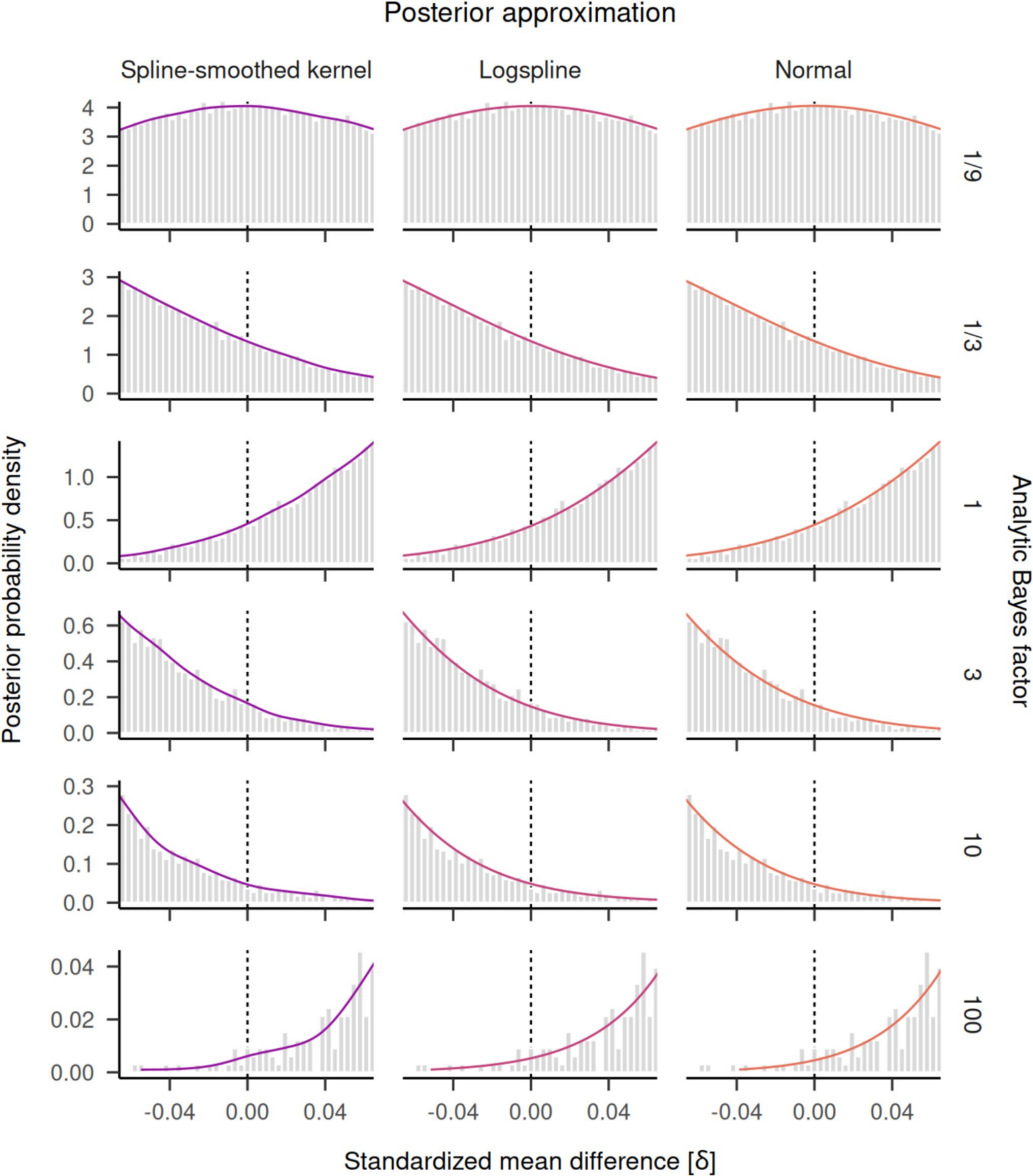
Histograms of 102,000 samples from the marginal posterior distribution for six standardized mean differences δ—implying JZS-Bayes factors between 1/9 and 100—and the approximate posterior probability density functions based on three different posterior density estimates

For the Savage–Dickey density ratios, the variability of estimates increased disproportionately with the evidence against the constrained model, ${\mathrm{BF}}_{10}$. Across all conditions, the spline-smoothed kernel estimates were more variable than the logspline estimates, which in turn were more variable than the normal approximation of the posterior densities. Figure 7 illustrates the reason for the differences in variance between the density approximation methods. Shown is one exemplary set of 102,000 posterior MCMC samples and the estimated density curves of each method. The normal distribution and the logspline density estimates yield relatively close, smooth fits; the spline-smoothed kernel density procedure yields a noticeably more jagged curve—especially in the tails of the distribution. Finally, Fig. 6 shows that, as expected, the conditional marginal density estimates were the least variable—the variance was 1–2 orders of magnitude smaller than for all other methods.

**Fig. 8:**
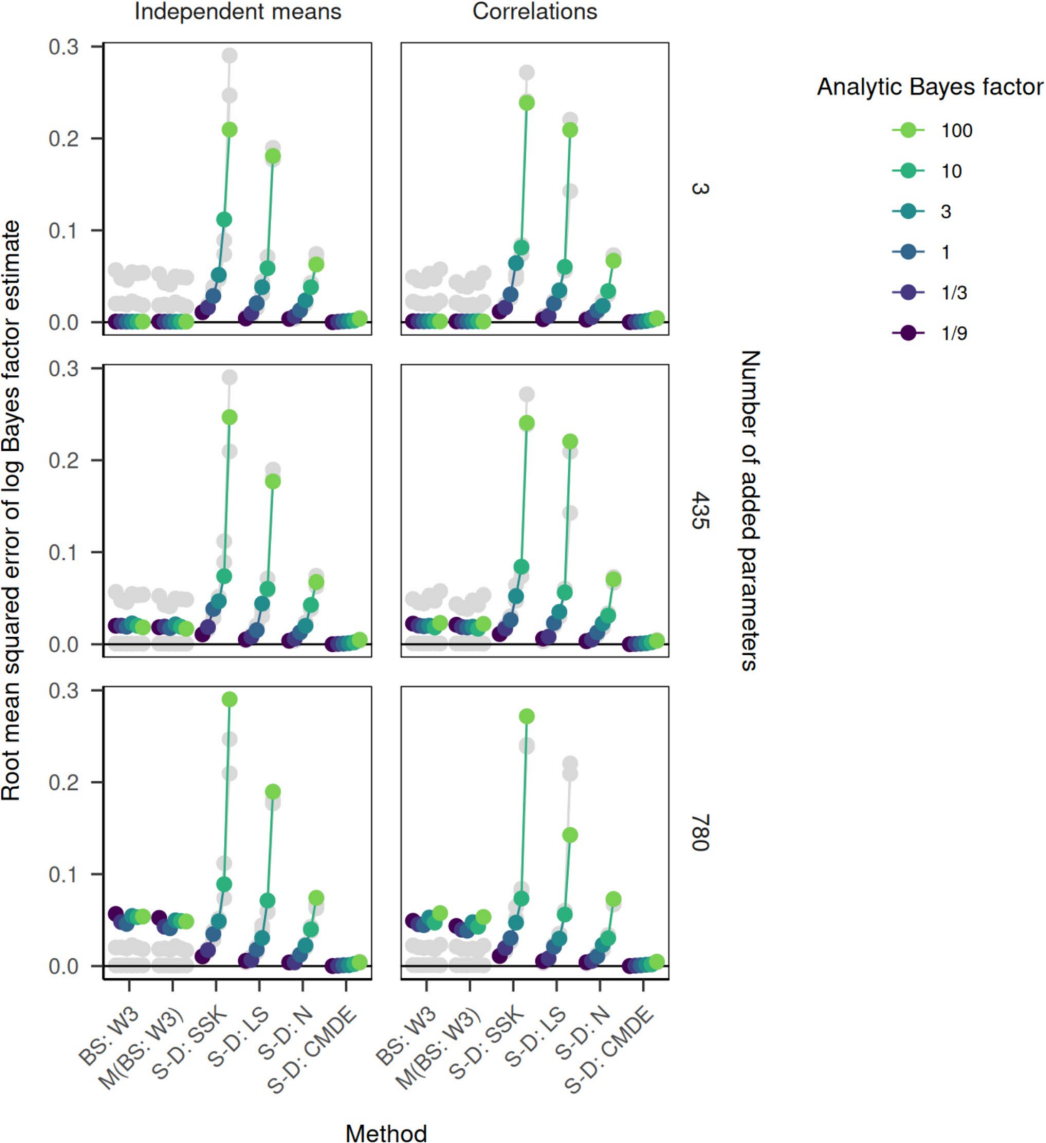
Root mean squared error of log Bayes factors estimated using the Warp-III bridge sampling algorithm in two variants (BS: W3 = 30 individual estimates for each of 30 sets of posterior samples; M(BS: W3) = means across the 30 individual estimates for each of 30 sets) or Savage–Dickey density ratio (S-D) based on four different posterior density estimates: Spline-smoothed kernel density (SSK), logspline density (LS), normal probability density (N), and conditional marginal density estimation (CMDE). Bayes factors were estimated for six datasets with analytic BF varying from 1/9 to 100. To vary the dimensionality of the posterior, we added 3, 435, or 780 parameters that were unrelated to the model likelihood. *Grey points* show results for different numbers of added parameters for comparison

Inspection of the bridge sampling results reveals two trends: First, the variability of the BF estimates was proportional to the true BF. For example, with 780 added parameters, the range of estimates was approximately ± 10%; see Figs. 4 and 6. Second, the variability of estimates increased substantially as the number of model parameters increased (Fig. 6). Increasing the number of added parameters from three to 780 increased the variability by almost two orders of magnitude. Averaging 30 repeated runs of the bridge sampling algorithm for any one set of MCMC samples reduced the variability of the BF estimates, albeit only modestly (Figs. 4 and 8). Averaging can only reduce variance attributable to the bridge sampling procedure, but not variance due to drawing a finite number of MCMC samples. Estimates of the two sources of variance from a hierarchical model indicated that in our simulation, the variance attributable to MCMC sampling was larger than the variance attributable to the bridge sampling algorithm. We have done a similar comparison in an empirical dataset, where the relative magnitudes of the two variance components were reversed: the variance due to the bridge sampling algorithm was larger. Averaging across several repeated runs of the bridge sampling algorithm will be more effective in situations like this. Unfortunately, it is currently unclear what determines the relative magnitudes of these variances.

Finally, a comparison of all estimation methods showed that bridge sampling was more reliable than all Savage–Dickey density ratios for models with only three added parameters in almost all conditions (Fig. 6). Only when the evidence favored the constrained model (${\mathrm{BF}}_{10}<1$) was the “best-case” conditional marginal density estimate more reliable than bridge sampling. For models with 780 added parameters, however, the Savage–Dickey methods were often more reliable, especially when ${\mathrm{BF}}_{10}\le 1$. With 780 added parameters, all estimates from the normal approximation were more reliable than bridge sampling. Clearly, with the simple model used here and a sample size of n=100 the posterior was well approximated by a normal distribution—under less favorable conditions, the normal approximation can be less accurate.

#### Overall estimation error

Our results show that the estimation methods differ in bias and reliability. For an integrative comparison of the methods, Fig. 8 shows each method’s root mean squared error (RMSE)—the average deviation of the estimated from the true log BFs. The “best-case” conditional marginal density estimate performed excellently in all conditions with a negligible RMSE. Bridge sampling estimates showed larger RMSE than the conditional marginal density estimates when we added 435 or 780 parameters to the model. Savage–Dickey density ratios using spline-smoothed kernel, logspline, or normal density estimates showed only slightly higher RMSE than bridge sampling for BFs in support of the constrained model (${\mathrm{BF}}_{10}\le 1$), that is, when the bulk of the posterior distribution was close to the test value ${\theta}_{0}$. Their performance was on par with or better than bridge sampling when we added 435 or 780 parameters to the model. Notably, for 780 added parameters, the Savage–Dickey density ratio based on the normal density estimates showed comparable RMSE to bridge sampling for all true BFs realized in our simulation. The spline-smoothed kernel and logspline density estimates exhibited substantially larger RMSE when the bulk of the posterior distribution was far away from the test value ${\theta}_{0}$. However, for 780 added parameters, they showed comparable RMSE to bridge sampling for true Bayes factors up to ${\mathrm{BF}}_{10}=10$. Nevertheless, with 102,000 MCMC samples, the absolute RMSE of all estimators was acceptable across all conditions of this simulation. This risk of an incorrect conclusion about the presence or absence of an effect was negligible.

#### Computational cost

A final aspect to consider when comparing BF estimation methods is their computational and implementation cost. As mentioned previously, we believe most researchers place a premium on ease of use. Conditional marginal density estimation is accurate and computationally cheap but can be difficult or impossible to implement. Hence, we do not consider it further. The remaining methods are easy to use but vary in accuracy and computational cost^16^.

**Fig. 9:**
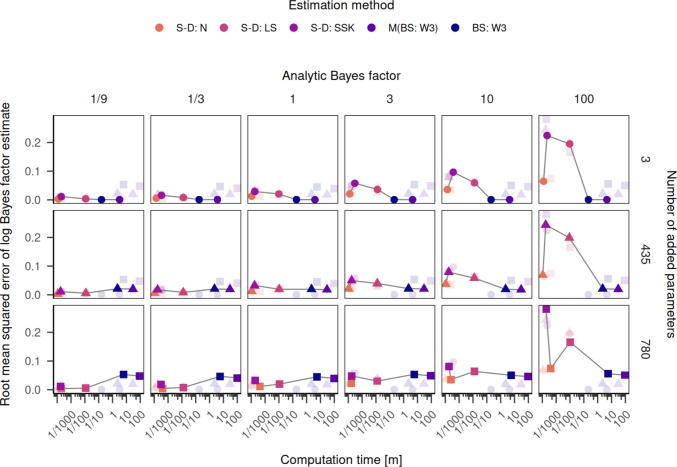
Comparison of computation time required to estimate the Bayes factor in minutes (*X*-axis on log_10_-scale, i.e., orders of magnitude) and root mean squared error of log Bayes factor estimates (*Y*-axis) for the Warp-III bridge sampling algorithm in two variants (BS: W3 = 30 individual estimates for each of 30 sets of posterior samples; M(BS: W3) = means across the 30 individual estimates for each of 30 sets) or Savage–Dickey density ratio (S-D) based on four different posterior density estimates: Spline-smoothed kernel density (SSK), logspline density (LS), and normal probability density (N). The bridge sampling computation was parallelized across six processor cores. Faint *points* show results for different numbers of added parameters for comparison

Figure 9 shows how RMSE relates to the time required to compute BF estimates. Savage–Dickey density ratios were computationally very cheap. The time required to compute the Spline-smoothed kernel and normal density estimates was negligible. Although logspline density estimation was two orders of magnitude slower, with 0.5 s, the computation time will be of no concern to the practical researcher. Because estimating the posterior density at the test value $\widehat{{f}_{\Theta }}\left(\theta =0\right)$ only requires the marginal posterior distribution of θ, the absolute computation time for the Savage–Dickey density ratio was largely unaffected by the number of model parameters—the minor increase in computation cost is caused by additional time required to extract the marginal posterior samples from the data object containing the joint posterior samples.

In contrast to the Savage–Dickey density ratios, bridge sampling estimates a model’s marginal likelihood for both the unconstrained and the constrained model. That is, estimating the BF requires posterior samples from both models. Hence, the computation time for bridge sampling includes the time required to sample from the constrained model. This cost increases with the number of model parameters.^17^ To speed up the computation, we parallelized the bridge sampling algorithm across six processor cores. With only three added model parameters, bridge sampling was approximately one order of magnitude slower than logspline density estimation—the slowest Savege-Dickey density ratio. With less than 10 s the computation time is noticeable but unlikely to deter the practical researcher. At 780 added model parameters, the computation time increased to approximately 5 min. Repeatedly running the bridge sampling algorithm 30 times on a set of MCMC samples further increased the computation time by approximately 1.5 orders of magnitude (Fig. 9). At 780 added model parameters, the computation time was approximately 100 minutes—despite parallel computation across six cores.

Finally, considering RMSE and computation time jointly reveals the return on invested computing time. Across all conditions, computation of the Savage–Dickey density ratio using the normal approximation was near instantaneous and very accurate. In this simulation, investing additional computation time to use more flexible posterior density estimation methods had a bad return on investment: Although the absolute computation cost was negligible, the RMSE *increased* compared to the normal approximation. Switching to bridge sampling increased computation time by 3–4 orders of magnitude with a modest decrease in RMSE under favorable conditions, i.e., few model parameters or at least moderate evidence against the constrained model, ${\mathrm{BF}}_{10}\ge 10$. For many model parameters or evidence in favor of the constrained model, the switch to bridge sampling had a bad return on investment: Despite the added computation time, the RMSE was often comparable or *increased* relative to the Savage–Dickey ratio using the normal approximation.

### Discussion

The results of this last simulation confirm that the variance of the Savage–Dickey density ratio increases disproportionately as the true BF increases. In contrast, the number of model parameters does not affect the Savage–Dickey density ratio but substantially increases the variance of bridge sampling estimates. What is more, whereas the number of model parameters has no appreciable effect on the computational cost of the Savage–Dickey density ratio, it further increases the cost of the bridge sampling algorithm. At first glance, the number of model parameters used in this simulation (437 and 782) may appear unreasonably large. However, hierarchical general linear models with multiple within-subject factors, random slopes, and a fair number of participants can involve even more model parameters. Our Simulation 4, for example, with 100 simulated participants, already included 812 parameters. However, even with 782 model parameters, the variance of the bridge sampling estimates in Simulation 6 was substantially smaller than in Simulation 4. We speculate that the lower variance reflects a closer fit between posterior and proposal distribution—a result of the simulation approach and the use of the Warp-III rather than the multivariate normal proposal distribution (Meng & Schilling, 2002). Bridge sampling is computationally more expensive and can be less reliable than the Savage–Dickey density ratio, but its estimates were unbiased in all conditions.

## General discussion

Across six simulations, we have explored reliability, bias, and computational cost of four easy to use methods of estimating BFs for testing whether an effect of an independent on a dependent variable exists or not. We have identified four key factors to consider when choosing between these methods: The number of MCMC samples, the number of model parameters, the magnitude of the true BF—or more precisely the tail-probability of the test value ${\theta}_{0}$ under the posterior distribution—and the shape of the posterior distribution.

We compared the performance of bridge sampling to the Savage–Dickey density ratio based on three commonly used posterior density approximations: the normal distribution, logspline density estimation (used in the R package bayestestr (Makowski et al., 2019), and spline-smoothed kernel density estimation (used in the R package brms (Bürkner, 2025). In broad strokes, bridge sampling is a flexible and accurate but computationally expensive approach; the Savage–Dickey density ratio is computationally much cheaper but less flexible and can be less accurate when the tail-probability of the test value ${\theta}_{0}$ in the posterior distribution is small. When choosing between bridge sampling and the Savage–Dickey density ratio, researchers should consider the computational cost of sampling from the posterior distribution, the number of model parameters, the strength of evidence, the number of model parameters to test, and the sample size.

We found the reliability of both estimation methods satisfactory. For most of the conditions we investigated—which we deem realistic for psychological experiments—the variability in BFs across multiple estimates with the same data is small in proportion to the strength of evidence. The few cases in which we observed substantial variability of BF estimates were cases in which the evidence for an effect was so strong that even large mis-estimations made no difference for the conclusion that there is unambiguous evidence for an effect. One exception to this assessment is when we used a large sample size (*N* = 2 x 100 in Simulation 4), leading to a high number of estimated parameters: In this case, the BF estimates from bridge sampling—but not those from the Savage–Dickey method—became variable to a degree that can mislead conclusions.

The reliability of BF estimates increases with the number of posterior MCMC samples used in the estimation (Gronau et al., 2019). If the data and model combine to make MCMC sampling cheap, and processing large data objects is no concern, bridge sampling is a good default. In our simulations, for bridge sampling 72,000 MCMC samples were sufficient to estimate a wide range of BFs with reasonable reliability in linear and logistic mixed-effects models with 2–3 independent variables and about 40 participants. However, more generally, we observed an unfavorable cost–benefit trend: As the number of model parameters increases, bridge sampling becomes computationally more expensive—both in absolute terms and relative to the Savage–Dickey density ratio—while the resulting BF estimates become less reliable. In contrast, we showed that the reliability of Savage–Dickey density estimates is unaffected by the number of model parameters, because only the marginal distribution of the to-be-tested parameter is estimated. In models with many parameters, such as hierarchical models with multiple random effects and many participants, Savage–Dickey density ratios are not only cheaper to compute but often just as reliable as, and sometimes more reliable than, bridge sampling. When choosing bridge sampling, researchers should use available diagnostics to assess the variance of bridge sampling estimates (Frühwirth‐Schnatter, 2004; Micaletto & Vehtari, 2025).

In our experience, it is often the case that computational cost is a concern—because MCMC sampling is very expensive or because there are many to-be-tested parameters. In this case, the Savage–Dickey density ratio is a good alternative to bridge sampling. We found that Savage–Dickey density ratios are often more variable than bridge sampling estimates and that, unlike bridge sampling estimates, their variability increases disproportionally with the strength of the evidence for an effect. Our simulations show that the reliability of Savage–Dickey density estimates is highest when the bulk of the posterior distribution is close to zero (i.e., the test value), implying that there is evidence for the absence of an effect. Conversely, if the bulk of the posterior distribution is far from 0, implying substantial evidence for an effect, the reliability of Savage–Dickey density ratios quickly decreases. The resulting risk of drawing incorrect conclusions can be mitigated by drawing enough MCMC samples. What is enough depends on the posterior density estimation method. Approximating the posterior distribution using a normal distribution resulted in the most efficient and least variable estimates. When the posterior distribution was normally distributed, we found that as few as 5000 uncorrelated MCMC samples can be enough. However, we also showed that the normal approximation can lead to substantial bias when the posterior distribution is decidedly non-normal. Logspline and spline-smoothed kernel density estimation make fewer assumptions about the distribution’s shape but require more MCMC samples. Our simulations show that these methods, too, can be biased, but in contrast to the normal approximation, this bias can be reduced by increasing the number of MCMC samples. Based on our simulations, we suggest that logspline density estimation requires at least 65,000 MCMC samples^18^ and spline-smooth kernel density estimation requires at least 100,000 MCMC samples.

In combination, the considerations above imply that the sample size—here, the number of participants—should also factor in the decision between BF estimation methods. In models with random effects, a larger sample size implies a larger number of (participant-level) model parameters, resulting in less reliable BF estimates from bridge sampling and higher computational costs. Moreover, larger samples often yield posterior distributions that are better approximated by normal distributions. Both these factors favor the Savage–Dickey density ratio over bridge sampling. Moreover, note that the relative performance of bridge sampling in our simulations could be considered optimistic. Researchers who have applied bridge sampling in their work may be aware that the bridge sampling algorithm can fail to converge and multiple attempts with different starting values may be necessary to estimate the marginal likelihood. These failures increase computational costs and can increase the variability of the estimates. In Simulation 6, which compared the methods on reliability and computational cost, the bridge sampler always converged quickly. In this sense, the results for bridge sampling may be considered optimistic.

Despite its advantages, the practical researcher should carefully consider whether the Savage–Dickey density ratio is applicable. The resulting estimate is equal to the desired BF only when in the constrained model the prior distribution on the shared parameters ϕ is equal to the prior in the unconstrained model *conditional* on the constraint, ${{p}_{{M}_{0}}\left(\phi \right)=p}_{{M}_{1}}\left(\phi \right| \theta ={\theta}_{0})$ (Dickey, 1971). For example, Heck (2019) showed that this assumption is violated in (hierarchical) linear models that use the Jeffrey-Zellner-Siow (JSZ) default priors, as, for example, implemented in the BayesFactor package (Morey & Rouder, 2015). Furthermore, the assumption can be violated when the to-be-tested parameter is computed from other model parameters rather than estimated directly. When in doubt, we recommend using bridge sampling.

Finally, testing the effects of predictor variables with more than two levels with the Savage–Dickey density ratio is challenging because it involves joint hypothesis tests of the presence or absence of multiple parameters. This requires a multidimensional generalization of the posterior density estimate required for the Savage–Dickey density ratio. We caution that these generalizations will require substantially larger numbers of MCMC samples to match the accuracy of the unidimensional case considered in our simulation—in particular, for spline-smoothed kernel and logspline density estimation (also see Morey et al., 2011, p. 371). A practical solution for testing independent variables with more than two levels is to break them down into orthogonal contrasts, each of which can be tested with the Savage–Dickey density ratio.

To conclude, our simulations provide practical guidance for choosing between easy-to-use and broadly applicable BF estimation methods, bridge sampling, and the Savage–Dickey density ratio. We have shown that both methods can produce sufficiently reliable and unbiased BF estimates in linear and logistic hierarchical models. The Savage–Dickey density ratio is computationally more efficient, especially for complex designs, because only one model needs to be run to extract the information needed for estimating BFs on all fixed effects in the design. In contrast, the bridge sampling method requires fitting every constrained model involved in the comparisons of interest. Hence, for Bayesian hypothesis testing within the generalized linear model framework, the Savage–Dickey density ratio offers a compelling combination of accuracy and computational efficiency to the practical researcher.

## Data Availability

Not applicable.

## Footnotes

1 The Bayesian information criterion is another easy-to-use method that can be used to approximate the Bayes factor (Delattre et al., 2014; Wagenmakers, 2007). We do not consider it here, because the BIC is inflexible with respect to the prior specification, as it has the assumption of a unit information prior built in.

2 The marginal prior distribution of the parameter of interest is usually available analytically. This is always the case if the model is parameterized such that a prior can be placed directly onto the parameter. However, even if the resulting distribution is not available analytically, it can usually be estimated efficiently because only samples from the marginal prior distributions on the constituent model parameters are required.

3 In such cases, the predictor could be decomposed into contrasts, each of which could be tested through the Savage–Dickey density ratio.

4 The normality assumption is often not justified for the individual data points—for instance, it is clearly violated for accuracy and response-time data—but can be justified by the central-limit theorem when aggregating over sufficiently large number of trials.

5 The specification of the subject-specific random intercept and random slope using the || indicates that random effects are assumed to be uncorrelated.

6 Note that the inclusion of all random effects drastically increases the number of to-be-estimated parameters in the model. For the specified model with 40 subjects, we are estimating a total of 87 parameters: the mean for one intercept, two main effects, and one interaction on the population level (4), the standard deviation for the intercept and the main effect of iv1 (2), 40 random participant effects on the intercept and main effect of iv1, respectively (80), and one parameter for the standard deviation on the residuals.

7 We computed the variance of the log-BF within each simulation, averaged the variances across simulations, then took the square root.

8 Large variability would be problematic even for large BFs if the ratio of two BFs were used to calculate a third. While this can be a valid concern, it is less relevant for the applications of the methods discussed here.

9 The full model now contains one intercept, three main effects and four interactions, and four of these eight effects vary within-subject. The random-effects structure in Simulation 4 omitted the interaction of IV1 and IV3 because with aggregated data, this random effect is confounded with measurement noise in the normal model. This leads to a total of 132 parameters for the normal model, and 131 for the binomial model.

10 This model comparison approach differs from the one implied by the Savage–Dickey density ratio. The Savage–Dickey density ratio is based on what is sometimes referred to as type III model comparisons: Every model term is tested by comparing the full model to a restricted model that removes only the to-be-tested term. Unless all model terms are orthogonal, that is, no portion of the to-be-explained variance can be explained by more than one model term, these two model comparison approaches will yield different results. We simulated orthogonal model terms by creating a balanced design and centering all predictors. Hence, both Bayes factors can be expected to be the same.

11 The total number of to-be-estimated parameters now increases to 812.

12 In brms, Savage–Dickey density ratios can be estimated via the density_ratio()-function—usually called indirectly via the function hypothesis().

13 Note that the distribution of the estimates becomes more skewed as the posterior tail probability decreases and is substantially more skewed than that of the logspline estimates. Hence, the bias of the spline-smoothed kernel estimates is *much more* severe when measured by the mean (expected value) of the distribution.

14 Only with three added parameters did we sometimes observe one divergent transition (out of 102,000 MCMC samples) on a few simulation runs. The divergent transitions appear to be related to the conditional marginal density estimate, which in practice would not be calculated. Results from these runs are not noticeably different from other runs.

15 Across all conditions, the bridge sampling algorithm failed to converge on nine simulation runs. These failures appear to be related to the conditional marginal density estimate, which are not required for bridge sampling. Hence, we excluded these simulation runs to avoid unduly inflating the variance of bridge sampling estimates.

16 This simulation was run on Desktop computers with six Intel(R) Core(TM) i5-8500 CPU and 32 GB RAM. Although computation time depends on the hardware and software used, the comparison between methods is informative beyond the specific hardware used here.

17 In hierarchical linear models, increasing the number of subjects (i.e., random effects) can substantially increases in the number of parameters. For these models the computation time “penalty” for fitting the constrained model will be more substantial than in this simulation because evaluation of the model likelihood will be slower. In this sense, bridge sampling computation times can be higher in practice than our simulation suggests.

18 The implementation of the logspline method in the R package logspline (Kooperberg, 2024) failed without warnings or errors when we submitted 500,000 posterior samples (see Simulation 5), which suggests that there is an upper bound of MCMC samples that this method can currently handle.

## Code availability

https://osf.io/wg8b4/

## References

[CR1] Bernardo, J. M., & Smith, A. F. M. (2000). *Bayesian theory*. Wiley.

[CR2] Bürkner, P.-C. (2025). Brms: An R package for Bayesian multilevel models using Stan. *Journal of Statistical Software,**80*, 1–28. 10.18637/jss.v080.i01

[CR3] Chib, S. (1995). Marginal likelihood from the Gibbs output. *Journal of the American Statistical Association,**90*, 1313–1321. 10.1080/01621459.1995.10476635

[CR4] Delattre, M., Lavielle, M., & Poursat, M.-A. (2014). A note on BIC in mixed-effects models. *Electronic Journal on Statistics, 8*. 10.1214/14-ejs890

[CR5] Dickey, J. M. (1971). The weighted likelihood ratio, linear hypotheses on normal location parameters. *The Annals of Mathematical Statistics,**42*, 204–223. 10.1214/aoms/1177693507

[CR6] Dickey, J. M., & Lientz, B. P. (1970). The weighted likelihood ratio, sharp hypotheses about chances, the order of a Markov chain. *The Annals of Mathematical Statistics,**41*, 214–226. 10.1214/aoms/1177697203

[CR7] Frühwirth-Schnatter, S. (2004). Estimating marginal likelihoods for mixture and Markov switching models using bridge sampling techniques*. *The Econometrics Journal,**7*(1), 143–167. 10.1111/j.1368-423X.2004.00125.x

[CR8] Gelfand, A. E., & Adrian, F. M. S. (1990). Sampling-based approaches to calculating marginal densities. *Journal of the American Statistical Association,**85*(410), 398–409. 10.2307/2289776

[CR9] Gelman, A., & Meng, X. M. (1998). Simulating normalizing constants: From importance sampling to bridge sampling to path sampling. *Statistical Science,**13*, 163–185. 10.1214/ss/1028905934

[CR10] Green, P. J. (1995). Reversible jump Markov chain Monte Carlo computation and Bayesian model determination. *Biometrika,**82*, 711–732. 10.1093/biomet/82.4.711

[CR11] Gronau, Q. F., Heathcote, A., & Matzke, D. (2020). Computing Bayes factors for evidence-accumulation models using Warp-III bridge sampling. *Behavior Research Methods,**52*(2), 918–937. 10.3758/s13428-019-01290-6 31755028 10.3758/s13428-019-01290-6 PMC7148283

[CR12] Gronau, Q. F., Sarafoglou, A., Matzke, D., Ly, A., Boehm, U., Marsman, M., Leslie, D. S., Forster, J. J., Wagenmakers, E.-J., & Steingroever, H. (2017). A tutorial on bridge sampling. *Journal of Mathematical Psychology,**81*, 80–97. 10.1016/j.jmp.2017.09.005 29200501 10.1016/j.jmp.2017.09.005 PMC5699790

[CR13] Gronau, Q. F., Singmann, H., & Wagenmakers, E. J. (2018). *bridgesampling: An R package for estimating normalizing constants*. arXiv, https://arxiv.org/pdf/1710.08162.pdf.

[CR14] Gronau, Q. F., Wagenmakers, E. J., Heck, D. W., & Matzke, D. (2019). A simple method for comparing complex models: Bayesian model comparison for hierarchical multinomial processing tree models using Warp-III bridge sampling. *Psychometrika,**84*, 261–284. 10.1007/s11336-018-9648-3 30483923 10.1007/s11336-018-9648-3 PMC6684497

[CR15] Heck, D. W. (2019). A caveat on the Savage–Dickey density ratio: The case of computing Bayes factors for regression parameters. *British Journal of Mathematical and Statistical Psychology,**72*(2), 316–333. 10.1111/bmsp.12150 30451277 10.1111/bmsp.12150

[CR16] Jeffreys, H. (1961). *The theory of probability*. Clarendon.

[CR17] Kooperberg, C. (2024). Package “logspline”. Retrieved from https://cran.r-project.org/web/packages/logspline/index.html

[CR18] Kooperberg, C., & Stone, C. J. (1991). A study of logspline density estimation. *Computational Statistics & Data Analysis,**12*, 327–347. 10.1016/0167-9473(91)90115-i

[CR19] Lee, M. D., & Wagenmakers, E.-J. (2014). *Bayesian modeling for cognitive science: A practical course*. Cambridge University Press.

[CR20] Llorente, F., Martino, L., Delgado, D., & López-Santiago, J. (2023). Marginal likelihood computation for model selection and hypothesis testing. An extensive review. *SIAM Review,**65*, 3–58. 10.1137/20m1310849

[CR21] Makowski, D., Ben-Shachar, M. S., Chen, S. H. A., & Lüdecke, D. (2019). Indices of effect existence and significance in the Bayesian framework. *Frontiers in Psychology*. 10.3389/fpsyg.2019.02767 31920819 10.3389/fpsyg.2019.02767 PMC6914840

[CR22] Meng, X.-L., & Schilling, S. (1996). Fitting full-information item factor models and an empirical investigation of bridge sampling. *Journal of the American Statistical Association,**91*, 1254–1267. 10.1080/01621459.1996.10476995

[CR23] Meng, X.-L., & Schilling, S. (2002). Warp bridge sampling. *Journal of Computational and Graphical Statistics,**11*(3), 552–586. 10.1198/106186002457

[CR24] Meng, X.-L., & Wong, W. H. (1996). Simulating ratios of normalizing constants via a simple identity: A theoretical exploration. *Statistica Sinica, 6*(4), 831–860. Retrieved from http://www.jstor.org/stable/24306045

[CR25] Micaletto, G., & Vehtari, A. (2025). Bridge sampling diagnostics. *arXiv, *arXiv:2508.14487. 10.48550/arXiv.2508.14487

[CR26] Morey, R. D., & Rouder, J. N. (2015). BayesFactor (Version 0.9.12.2). Retrieved from http://cran.at.r-project.org/web/packages/BayesFactor/index.html

[CR27] Morey, R. D., Rouder, J. N., Pratte, M. S., & Speckman, P. L. (2011). Using MCMC chain outputs to efficiently estimate Bayes factors. *Journal of Mathematical Psychology,**55*, 368–378. 10.1016/j.jmp.2011.06.004

[CR28] Oberauer, K. (2022). The importance of random slopes in mixed models for Bayesian hypothesis testing. *Psychological Science,**33*(4), 648–665. 10.1177/09567976211046884 35357978 10.1177/09567976211046884

[CR29] R Core Team. (2024). R: A language and environment for statistical computing (Version 4.4.2). Vienna, Austria: R Foundation for Statistical Computing. Retrieved from URL: http://www.R-project.org

[CR30] Rouder, J. N., Morey, R. D., Speckman, P. L., & Province, J. M. (2012). Default Bayes factors for ANOVA designs. *Journal of Mathematical Psychology,**56*, 356–374.

[CR31] Rouder, J. N., Speckman, P. L., Sun, D., & Morey, R. D. (2009). Bayesian *t* tests for accepting and rejecting the null hypothesis. *Psychonomic Bulletin & Review,**16*, 225–237. 19293088 10.3758/PBR.16.2.225

[CR32] Sinharay, S., & Stern, H. S. (2005). An empirical comparison of methods for computing Bayes factors in generalized linear mixed models. *Journal of Computational and Graphical Statistics,**14*, 415–435. 10.1198/106186005x47471

[CR33] Stan-Development-Team. (2024). *Stan Modeling Language Users Guide and Reference Manual, Version 2.35*. Retrieved from https://mc-stan.org

[CR34] Wagenmakers, E.-J. (2007). A practical solution to the pervasive problems of p values. *Psychonomic Bulletin & Review,**14*, 779–804. 18087943 10.3758/bf03194105

[CR35] Wagenmakers, E.-J., Lodewyckx, T., Kuriyal, H., & Grasman, R. P. P. P. (2010). Bayesian hypothesis testing for psychologists: A tutorial on the Savage–Dickey method. *Cognitive Psychology,**60*, 158–189. 20064637 10.1016/j.cogpsych.2009.12.001

[CR36] Wasserman, L. (2000). Bayesian model selection and model averaging. *Journal of Mathematical Psychology,**44*, 92–107. 10733859 10.1006/jmps.1999.1278

[CR37] Wetzels, R., Raaijmakers, J. G. W., Jakab, E., & Wagenmakers, E.-J. (2009). How to quantify support for and against the null hypothesis: A flexible WinBUGS implementation of a default Bayesian *t*-test. *Psychonomic Bulletin & Review,**16*, 752–760. 19648463 10.3758/PBR.16.4.752

